# Gasotransmitters and noble gases in cardioprotection: unraveling molecular pathways for future therapeutic strategies

**DOI:** 10.1007/s00395-024-01061-1

**Published:** 2024-06-15

**Authors:** Pasquale Pagliaro, Nina C. Weber, Saveria Femminò, Giuseppe Alloatti, Claudia Penna

**Affiliations:** 1 Department of Clinical and Biological Sciences, University of Turin, Regione Gonzole 10, 10043 Orbassano, TO) Italy; 2 National Institute for Cardiovascular Research (INRC), 40126 Bologna, Italy; 3 Department of Anesthesiology, Laboratory of Experimental Intensive Care and Anesthesiology-L.E.I.C.A, Amsterdam University Medical Centers, Amsterdam Cardiovascular Science (ACS), Amsterdam, The Netherlands; 4 Uni-Astiss, Polo Universitario Rita Levi Montalcini, Asti, Italy

**Keywords:** Cardioprotective pathways, Noble gases, Gasotransmitters, Reactive oxygen species, Mitochondrial permeability transition pore

## Abstract

Despite recent progress, ischemic heart disease poses a persistent global challenge, driving significant morbidity and mortality. The pursuit of therapeutic solutions has led to the emergence of strategies such as ischemic preconditioning, postconditioning, and remote conditioning to shield the heart from myocardial ischemia/reperfusion injury (MIRI). These ischemic conditioning approaches, applied before, after, or at a distance from the affected organ, inspire future therapeutic strategies, including pharmacological conditioning. Gasotransmitters, comprising nitric oxide, hydrogen sulfide, sulfur dioxide, and carbon monoxide, play pivotal roles in physiological and pathological processes, exhibiting shared features such as smooth muscle relaxation, antiapoptotic effects, and anti-inflammatory properties. Despite potential risks at high concentrations, physiological levels of gasotransmitters induce vasorelaxation and promote cardioprotective effects. Noble gases, notably argon, helium, and xenon, exhibit organ-protective properties by reducing cell death, minimizing infarct size, and enhancing functional recovery in post-ischemic organs. The protective role of noble gases appears to hinge on their modulation of molecular pathways governing cell survival, leading to both pro- and antiapoptotic effects. Among noble gases, helium and xenon emerge as particularly promising in the field of cardioprotection. This overview synthesizes our current understanding of the roles played by gasotransmitters and noble gases in the context of MIRI and cardioprotection. In addition, we underscore potential future developments involving the utilization of noble gases and gasotransmitter donor molecules in advancing cardioprotective strategies.

## Introduction

Coronary artery disease persists as a major contributor to worldwide mortality and morbidity, driving extensive research aimed at safeguarding the myocardium from ischemic damage. Ischemic preconditioning (PC), postconditioning (PostC) and remote conditioning (RC) are extensively studied adaptive mechanisms. These procedures involve brief exposure to ischemia/reperfusion, occurring prior (PC) to prolonged ischemia, at the immediate onset of reperfusion (PostC), or in a remote organ (RC), respectively, thereby initiating cardioprotection. Yet, pharmacological conditioning, achieved through specific drug administration, can mimic the effects of ischemic PC and PostC, providing an alternative approach to heart protection. Conditioning protection is evidenced by notable reductions in arrhythmias, decreased infarct size, and alleviated cardiac and endothelial dysfunction [e.g., see Refs. 24, 60, 61, 64, 67, 68]. While the cardioprotective benefits of ischemic or pharmacological conditioning strategies have been demonstrated across various species, including humans, the presence of cardiovascular risk factors, comorbidities, and associated medications (comedications) may disrupt cardioprotective signaling pathways [5, 44, 92, 154]. In addition, the potential cardioprotective effect of female sex, with differences possibly existing between pre- and post-menopausal women, should be considered. However, there is currently no evidence indicating sex-related disparities in infarct size and cardioprotection in pigs [93, 106].

Nevertheless, the precise cellular mechanisms underlying the cardioprotective pathways remain elusive in male and female, although several signal transduction cascades have been proposed. The initiation of cardioprotective modalities involves mainly the occupancy of specific surface receptors by various ligands, leading to intracellular signaling transduction, including redox signaling by reactive oxygen species (ROS) [7, 69, 144, 152], S-nitrosylation by nitric oxide (NO) and its derivatives, and S-sulfhydration by hydrogen sulfide (H_2_S) [5, 7, 144]. These modalities interact and regulate an integrated pathway, impacting one another’s function. Notably, enzymes may undergo phosphorylation and/or nitrosylation at specific or distinct sites, resulting in alterations in their activity. The cardioprotective pathways, namely the survivor activating factor enhancement (SAFE) pathway, the reperfusion injury salvage kinase (RISK) and the NO/cyclic 3′,5′-guanosine monophosphate (cGMP)/protein kinase G (PKG) pathway, were initially associated with ischemic conditioning [see Refs. 91, 93, 95, 226]. Enhanced understanding of these pathways holds promise for advancing cardioprotective strategies, including pharmacological interventions, in clinical practice. In particular, we outline the elements of RISK pathway involving intracellular mediators such as phosphatidyl-inositol-4,5-bisphosphate 3-kinase (PI3K), protein kinase C (PKC), mitogen-activated protein kinase (MAPK), glycogen synthase kinase-3β (GSK-3β), and extracellular signal-regulated kinase 1/2 (ERK1/2), as well as the SAFE pathway influenced by janus kinase-signal transducer and activator of transcription (STAT) pathways [24, 226]. Convergence between these pathways occurs at various steps but mainly on the mitochondria and, specifically, on the mitochondrial permeability transition pore (mPTP) [24, 60, 61, 226]. Indeed, these factors and organelles represent a direct or indirect target for various gases, which exhibit biological effects, and among them, oxygen (O_2_) and NO, along with their derivatives, ROS and reactive nitrogen species (RNS), are well known for their biological activities.

For approximately four decades, it has been recognized that our organism possesses an enzymatic apparatus capable of generating gases with distinct biological effects. Termed collectively as gasotransmitters, including NO, H_2_S, sulfur dioxide (SO_2_) and carbon monoxide (CO), these gases constitute a group of endogenous, highly reactive, and regulatory molecules. As we will see in the specific paragraphs gases play a crucial role not only as endogenous factors, but also in cardioprotection induced by pharmacological conditioning using exogenous gasotransmitters as well as noble gases. Indeed, noble gases, traditionally labeled as ‘inert gases’, have gained recognition more recently due to their manifestation of distinctive biological effects.

Here, we focus on the cardioprotective effects of gasotransmitters and noble gases, allocating only some discussion to the role of ROS/RNS. The cardioprotective effects of ROS/RNS have been comprehensively reviewed by our group and others, emphasizing the intricate signaling mechanisms involved in cardioprotection; for reviews on the role of ROS/RNS in cardioprotection, see Refs. [7, 69, 144, 152]. In addition, some volatile anesthetics are gaseous molecules known for their protective effects [12, 203, 229]. Specifically, this review aims to provide insights into the roles of gasotransmitters and noble gases in myocardial ischemia/reperfusion injury (MIRI) and cardioprotection, while also discussing potential future directions for developing agents that modulate these molecules for cardioprotective purposes. The literature search was performed using the PubMed databases, employing as keywords the distinct gases in combination with conditioning and cardioprotection. By curating recent (last 20 years) English-language publications, we present an overview of current research and anticipate innovative approaches to enhance the effectiveness and broaden the clinical applications of cardioprotection.

## The vital gas, oxygen, in ischemia/reperfusion and cardioprotection: focus on supersaturated-oxygen (SSO_2_)

Given the vital role of O_2_ as a fundamental gas for life and as a component of certain gasotransmitters, it is necessary to acknowledge its significance not only for life but also for potential therapeutic applications. Therefore, before exploring the cardioprotective effects of gasotransmitters and noble gases in the context of MIRI and cardioprotection, we briefly discuss O_2_, considering both its essentiality and its detrimental or therapeutic paradox, such as the concept of SSO_2_ therapy.

The regulatory influence of O_2_ extends from embryonic ontogeny to pathological processes, making it a key participant in various cellular activities [112]. Oxygen is a crucial element for the survival of all mammals, playing a fundamental role in generating biological energy required by cells [112]. Fluctuating O_2_ levels influence cellular physiology, with evidence suggesting that variable O_2_ levels, rather than persistently low levels, pose the greatest harm [112].

The “oxygen paradox” arises from the dual role of O_2_ in both MIRI and cardioprotection [7, 144, 152, 203]. This paradox involves the harm caused by reoxygenation following ischemia, known as reperfusion injury, juxtaposed with the therapeutic potential of slow release of O_2_ [43, 133, 150] and gentle reperfusion [122] or super-reoxygenation [113, 190, 230]. In the context of ischemia/reperfusion, “*compartment syndrome*” refers to increased pressure within tissue compartments, impeding blood flow and causing potential damage [34, 110]. Although the term “*compartment syndrome*” is typically applied to peripheral muscle reperfusion injury rather than the heart, we employ it here to underscore the significant impact of edema on O_2_ delivery during reperfusion in the setting of MIRI [34, 48]. SSO_2_ has emerged as a potential therapeutic approach to mitigate MIRI-associated damage, particularly in addressing “*compartment syndrome*” [96, 230]. SSO_2_ is obtained through aqueous oxygen, creating a metastable saline solution with a higher concentration of dissolved O_2_ than the liquid carrier [191–193]. Catheter-delivered SSO_2_ serves as a pragmatic alternative to traditional hyperbaric oxygen therapy (HBOT) in critical care settings.

Research indicates that SSO_2_, similar to HBOT, assists in arterial vasoconstriction, addressing *compartment syndrome* without the risks associated with high gas-phase [O_2_]/tissue interfaces [191–193]. Preclinical studies demonstrate that intracoronary SSO_2_ infusion significantly reduces infarct size and the edematous area at risk in post-ST-segment elevation MI (STEMI) reperfusion scenarios [191–193].

The primary mechanism contributing to SSO_2_-induced cardioprotection is the acute improvement of microvascular flow, leading to the normalization of left ventricle ejection fraction [193]. SSO_2_ facilitates gradual reperfusion at relatively low pressures, reducing microvascular damage compared to abrupt reperfusion [177, 178]. The hyperosmotic effect of hyperbaric dissolved O_2_, coupled with reduced capillary hydrostatic pressure, aids in removing edema fluid through Starling’s principle, interrupting the cycle of MIRI and microvascular responses [177, 191–193].

SSO_2_ also addresses the inflammatory responses associated with MIRI, as evidenced by reduced myeloperoxidase levels in animal models [86, 191]. The resolution of the “oxygen paradox” is attributed to SSO_2_’s positive effects on microvascular flow, improvement of left ventricular function, and reduction of infarct size [10, 190]. The time course of SSO_2_-induced improvements, including the reduction of precapillary resistance, remains to be elucidated. In summary, SSO_2_ shows promise as a therapeutic intervention in MIRI scenarios by effectively addressing oxygen-related complications and inflammatory responses. Hence, here we see “the paradox of paradox”: the detriment posed by reoxygenation following ischemia (i.e., reperfusion injury), are counteracted by the therapeutic potential of SSO_2_. Alternative experimental strategies to mitigate the adverse effects associated with the oxygen paradox include stuttering and slow reperfusion [177, 183], or controlled O_2_ release by oxygen-loaded nanodevices. These nanodevices can act as biocompatible drug carriers, enabling the gradual release of O_2_, potentially along with therapeutic agents [43, 132, 150] (see also “Nanodevices to deliver gases”).

While preclinical and some clinical evidence suggest the potential improvement of MIRI with SSO_2_, it is essential to consider the implications of hyperbaric oxygen therapy, which may exacerbate heart failure [174], and it is advised to avoid hyperoxia in cases of chronic cyanosis [121]. Moreover, a meta-analysis has revealed weak and inconsistent evidence, along with modest statistical power, regarding the safety and efficacy of oxygen therapy [88]. Therefore, caution is warranted in the use of O_2_ in these contexts. Adequately powered studies are imperative to gain a better understanding of the role of SSO_2_ in patients undergoing coronary revascularization.

## Biological gasotransmitters

The intricate signaling pathways in which gasotransmitters—CO, H_2_S, SO_2_ and NO—are involved represent a multifaceted network of interactions characterized by the high reactivity and diffusive capacity inherent to these gaseous molecules. The intricacy of these pathways stems from both their chemical properties and the diverse cellular responses they modulate in the dynamic cardiovascular environment.

Gasotransmitters are versatile signaling mediators. They play a pivotal role in orchestrating cellular reactions critical to the complexity of cell physiology and pathophysiology, including MIRI [9, 112, 200, 220]. Their presence is integral to the delicate equilibrium between the potentially detrimental effects of ischemia and reperfusion. For instance, as signaling molecules, CO, H_2_S, SO_2_ and NO engage in a sophisticated crosstalk with cellular components, influencing vasodilation, inflammation, apoptosis, and oxidative stress [87].

The high reactivity of gasotransmitters and the capacity to rapidly traverse cellular membranes facilitate their widespread influence on various cellular components. Their diffusive capacity enables them to permeate tissues and reach subcellular compartments. Indeed, gasotransmitters, as well as derivative, ROS and RNS, are considered among the major factors involved in signaling pathways regulating mitochondrial function involved in cardioprotection [113, 200, 220].

As researchers explore gasotransmitter-mediated cellular responses, we expect this summary to be a valuable resource for understanding the complex dynamics involved in cardioprotection. A summary of the experimental studies on gasotransmitter-induced protection can be found in Tables 1, 2, 3 and 4.

**Table 1:** Overview of experimental studies on nitric oxide induced protection

Model	End point	Nitric oxide			
		Treatment	Targets	Results	Refs.
In vivo					
Gottinger miniswine (nitrous oxide and enflurane-anesthetized) (NRW/NRA)	Infarct size and subendocardial blood flow modification by PC	Ischemic PC and l-nitro arginine inhibition of NO synthase	NO synthesis inhibition	Significant reduction in infarct size and subendocardial flow by IPC, unaffected by NO inhibition. Endogenous NO is not involved in the classical IPC	[158]
Male and female mice and primary cardiomyocyte with specific NO-GC deletion (NRW/NRA)	Evaluation of the infarct-limiting effects of the endogenous NO-GC in vivo and cardiomyocyte protection	MIRI; in vivo model of AMI; ischemic PostC, sildenafil, tadalafil, cinaciguat, NS11021 (large conductance and Ca^2+^-activated potassium channel opener)	NO-GC, cGMP, large conductance and Ca^2+^-activated potassium channel	Lack of NO-GC in cardiomyocytes led to mild increase in blood pressure, and no effect on basal infarct sizes after I/R; PostC, sildenafil, tadalafil, cinaciguat significantly reduced infarction; NS11021 protected hearts in both NO-GC proficient and deficient mice	[46]
Mice in vivo Mb^−/−^ and eNOS^−/−^, weighing 32 ± 6 g and humans (male) healthy volunteers (NRW/NRA)	Evaluation of mitochondrial respiration, ROS formation, and myocardial infarct size	RIPC: 4 cycles of no-flow ischemia with reactive hyperemia	Circulating nitrite, eNOS, Mb, mitochondrial respiration, ROS, infarct size	Shear stress-stimulated eNOS and released nitric oxide, converted to nitrite, transferred to myocardium. Nitrite reduction by Mb reduces ROS, infarct size. NOS inhibition abrogated RIPC cardioprotection. Plasma from RIPC volunteers has cardioprotective nitrite effect	[168]
C57BL/6 J mice and nNOS KO, male, 9 to 10 weeks old (NRW)	Role of β3-AR and downstream eNOS/nNOS isoforms in heart failure and hypertrophy induced by TAC	TAC, BRL (0.1 mg/kg/h), or both	β3-AR, NOS, eNOS, nNOS	BRL attenuated TAC-induced left ventricular dysfunction, and cardiac hypertrophy. Increased NO production and reduced superoxide. TAC led to eNOS uncoupling, not reversed by BRL. BRL up-regulated protective nNOS expression, but its effects were detrimental in nNOS^(−/−)^ mice	[126]
Ex vivo/in vitro					
Isolated rat hearts perfused at constant flow or pressure obtained from Male Wistar rats, weighing 450–550 g	Role of the NO-cGMP pathway in infarct size reduction by PostC at the onset of reperfusion	30-min global ischemia and 120-min reperfusion; PostC; inhibition of NOS or GC	NO-cGMP pathway; cGMP release	PostC reduced infarct size and cGMP release	[149]
Isolated hearts perfused at constant flow obtained from Male Wistar rats, weighing 450–550 g	Role of BK B2 receptor and downstream pathway in infarct size reduction by PostC	PostC: 5 cycles of 10 s reperfusion/ischemia; intermittent BK	B_2_ BK receptors, NOS, PKG, mitoKATP channels	PostC significantly reduced infarct size. Protection abolished by B_2_ BK receptor antagonists, NOS inhibitor, PKG blocker, and mitoKATP blocker	[151]
Male and female hearts of C57BL/6 J mice, 12–16 weeks old (NRW)	Role of protein SNO on heart performance by adenosine A1 receptor by PPC and by IPC	Pharmacological preconditioning with CHA (adenosine A1 receptor agonist)	Nitric oxide, p-Akt, p-eNOS, SNO	Pharmacological CHA preconditioning improved functional recovery in both male and female hearts. Increased p-Akt and p-eNOS levels. CHA induced a modest increase in protein SNO levels in both male and female hearts compared to baseline	[179]
Isolated perfused hearts from male Sprague–Dawley rats, weighing 300 to 350 g	Role of PKG activity in ischemic PostC reduction of oxidative stress, eNOS uncoupling and infarct size	Ischemia/reperfusion and ischemic PostC	cGMP, PKG PI3K/Akt (RISK), eNOS	PostC reduced infarct size and cGMP depletion. Inhibition of cGMP synthesis, PKG, or NOS abolished protection	[81]
Isolated perfused hearts from Male Wistar rats, weighing 400–500 g	Evaluation of cardioprotective pathway of HNO and NO donors in reduction of infarct size	IPC; NO donor (DEA/NO); HNO donors (AS, or IPA/NO)	PKCε translocation, mitoKATP channel activation, mPTP opening	AS and IPA/NO, like IPC, reduced infarct size. DEA/NO was less effective. PKCε inhibitor prevented IPA/NO protection. HNO anti-ischemic effects were insensitive to mitoKATP channel inhibitor (5-HD). HNO donors inhibited mPTP opening, requiring PKCε translocation, not mitoKATP channel activation	[113]
Isolated perfused hearts obtained from Male Wistar rats, weighing 450–550 g	Evaluation of the infarct size reduction and post-ischemic heart performance by HNO and NO donors	HNO donor (AS), NO donor (DEA/NO), vehicle, buffer	Myocardial contractility, LDH release, infarct size	AS and IPC reduced infarct size and LDH release, and improved heart performance. DEA/NO was less effective. Nitroxyl scavenger reversed AS effects. HNO affords myocardial protection similar to IPC, greater than NO. Reactive nitrogen oxide species trigger protection via pro-oxidative and/or nitrosative stress-related mechanisms	[143]
Isolated perfused hearts obtained from male Sprague–Dawley rats, weighing 250–300 g	Contribution of mitoKATP channel in infarct size and improvement of heart performance in preconditioning by isoflurane	Preconditioning with isoflurane, DZ (5-HD), adenosine, SNAP	MitoKATP channel activity, infarct size, heart performance	Isoflurane increased mitoKATP channel activity, reducing infarct size. Adenosine and SNAP, combined with isoflurane, further reduced infarct size and improved heart performance. MitoKATP channel activation triggers cardioprotection. Combined preconditioning involves both mitoKATP channel-dependent and -independent mechanisms	[207]
Hearts isolated from sedentary or exercised Wistar male rats, weighing 382 ± 38 g	Evaluation of protective role of eNOS after exercise in terms of mitochondrial function	I/R with or without NOS inhibitors (L−NAME or L−NIO) or BH_4_	eNOS, eNOS-PSer1177, eNOS dimerization, SNO, nitro-oxidative stress and mPTP opening	Exercise protected hearts through eNOS, despite altered eNOS-PSer1177 and coupling during reperfusion. Increased SNO and reduced nitro-oxidative stress in exercised hearts were observed, and BH4 treatment reversed exercise-induced protection, highlighting the crucial role of eNOS uncoupling	[40]
Hearts isolated from New Zealand white rabbits of either sex (NRW/NRA)	Evaluation of endogenous and exogenous nitric oxide effects on infarct size	IPC with 5-min ischemia/10-min reperfusion or SNAP, a NO donor. Chelerythrine ( PKC blocker) with or shortly after SNAP, L-NAME (NOS blocker)	Cardioprotection by nitric oxide ROS and PKC	IPC or SNAP reduced myocardial infarct size. Chelerythrine and N-(2-mercaptopropionyl)-glycine (free radical scavenger) blocked SNAP’s cardioprotection. L-NAME failed to block the cardioprotection from the IPC protocol	[123]

*5-HD* 5-hydroxydecanoate; Akt/PKB protein kinase B;* AMI* acute myocardial infarction; AS, Angeli’ salt (Na_2_N_2_O_3_); BH4, tetrahydrobiopterin; BRL, BK, bradykinin; BRL37344 β3 agonist; cGMP, cyclic guanosine monophosphate; CHA, N6-cyclohexyl adenosine; DEA/NO, diethylamine/NO; DZ, diazoxide; eNOS, endothelial NOS; eNOS-PSer1177, eNOS phosphorylation at serine1177;* GC*, guanylyl cyclase; HNO, nitroxyl;* I/R* ischemia–reperfusion; IPA/NO, isopropylamine/NO; IPC, ischemic preconditioning;* LDH* lactate dehydrogenase; L-NAME, N-nitro-L-arginine methyl ester; L-NIO, N5-(1-iminoethyl)-L-ornithine; Mb, myoglobin; MIRI, myocardial ischemia/reperfusion injury; mitoKATP channel, mitochondrial K(ATP) channel; mPTP, mitochondrial permeability transition pore; nNOS, neuronal NOS; NO, Nitric Oxide; NOS, nitric oxide synthase; NRA, no reported age; NRW, no reported weight; PI3K, phosphatidylinositol-3-kinase;* PKC* protein kinase C; PKCε, protein kinase Cε;* PKG* Protein Kinase G; PostC, postconditioning; PPC, pharmacological PC; RIPC, remote ischemic preconditioning; RISK, reperfusion injury salvage kinase pathway; ROS, reactive oxygen species; SNAP, S-nitroso-N-acetyl-penicillamine; SNO, protein S-nitrosylation; TAC, Transverse aortic constriction; β3-AR, β3-adrenergic receptor

**Table 2:** Overview of experimental studies on hydrogen sulfide-induced protection

Hydrogen sulfide					
Model	Endpoint	Treatment	Targets	Results	Refs.
In vivo					
Yorkshire pigs of either sex, weighing 35–40 kg	Evaluation of exogenous hydrogen sulfide capacity to reduce infarct size, improve heart performance and to reduce inflammation	MIRI with placebo (Controls) or sulfide treatment	Arterial pressure, segmental shortening, infarct size, IL-6, IL 8, TNF-α, myeloperoxidase activity, coronary microvascular reactivity	Sulfide treatment improved post-ischemia mean arterial pressure and left ventricular pressure over time. It reduced infarct size, myeloperoxidase activity, and proinflammatory cytokines. Coronary microvascular reactivity was also enhanced in treated animals	[189]
CSE Knockout Mice eNOS phosphomutant mice (S1179A and Wild Type, Male mice 14–16 weeks old (NRW)	Evaluation of oxidative and nitrosative stress and infarct size in CSE KO mice with reduced H_2_S or NO levels	I/R with or without H_2_S therapy before reperfusion	eNOS, NO production, I/R injury	CSE knockout mice show elevated oxidative stress, dysfunctional eNOS, reduced NO, and exacerbated I/R injury. Acute H_2_S therapy restores eNOS function, enhances NO, and attenuates I/R injury. H_2_S therapy was ineffective in S1179A mice	[89]
Apolipoprotein E Knockout Mice, Wild type (WT) C57BL/6 mice (control), 6–8 weeks old (NRW)	Evaluation if H_2_S can promote plaque stability and the potential underlying mechanisms	NaHS or pravastatin daily for 14 weeks	Plaque stability, fibrous cap thickness, collagen content, blood lipid levels, plaque formation, VSMCs apoptosis, MMP-9 expression	NaHS enhanced plaque stability and protected against atherogenesis by decreasing VSMCs apoptosis and MMP-9 expression. Pravastatin increased fibrous cap thickness, reduced VSMC apoptosis, unaffected plaque collagen and MMP-9	[222]
Male Wistar rats, weighing 150 to 180 g	Role of H_2_S in the pathogenesis of HPH	Exogenous supply of H_2_S in HPH rat model	H_2_S, CO/HO pathway (HO-1 protein, HO-1 mRNA)	H_2_S significantly decreased in HPH. Plasma CO, HO-1 protein, and HO-1 mRNA increased. Exogenous H_2_S alleviated pulmonary arterial pressure elevation, increased plasma CO, and up-regulated HO-1 protein and mRNA in pulmonary arteries	[164]
Male Sprague–Dawley rats, 8 to 10 weeks old (NRW)	Evaluation of the protective effect of slow-releasing H_2_S donor GYY4137 on myocardial I/R injury (infarct size and heart performance) and possible signaling mechanisms	GYY4137 (12.5, 25 or 50 mg/kg/day) a slow-releasing H_2_S donor for 7 days	CSE activity, malondialdehyde, myeloperoxidase, superoxide anion, phospho-MAPK, Bcl-2, Bax, caspase-3, apoptosis	GYY4137 improved heart performance, reduced ischemic area, alleviated histological injury, and decreased plasma creatine kinase after myocardial I/R. It enhanced H_2_S concentration and CSE activity, attenuated oxidative stress, suppressed MAPK phosphorylation, and modulated apoptotic markers	[118]
Ex vivo/in vitro					
Isolated perfused mice hearts obtained from Male C57BL/6 J, 12–16 weeks old (NRW)	Evaluation of heart performance and infarct size and role of protein-SSH or -SNO by H_2_S and/or NO donors	NaHS (H_2_S donor, 100 µM) or SNAP (NO donor, 10 µM) or SNAP + NaHS	SSH, SNO, heart performance, infarct size	NaHS or SNAP reduced infarct size and improved heart performance. Combined treatment had an additive protective effect. C-PTIO (NO scavenger) eliminated protection by NaHS and SNAP + NaHS, indicating NO involvement. NaHS increased both SSH and SNO. Combo-treatment resulted in an additive increase in SNO, not SSH	[198]
Human microvascular endothelial cells-1	Ischemia/reperfusion injury (cell viability) and angiogenesis (cell migration)	NaHS (H_2_S donor, 5 mM) pretreatment for 24 h SCH772984 (Aurogene a ERK1/2 inhibitor, 1 µM)	ERK1/2 activation, mitochondrial function, cell viability, migration	H_2_S preconditioning enhanced cell viability and endothelial cell migration, while preserving mitochondrial function. Inhibition of ERK1/2 phosphorylation confirmed H_2_S role in this pathway	[238]

Bcl-2, B-cell lymphoma-2; Bax, Bcl-2-associated protein x; CO, carbon monoxide; C-PTIO, 2-(4-carboxy-2-phenyl)-4,4,5,5-tetramethylimidazoline-1-oxyl-3-oxide; CSE, cystathionine γ-lyase; eNOS, endothelial nitric oxide synthase; ERK1/2, extracellular signal-regulated kinase 1/2; H_2_S, hydrogen sulfide; HO-1, heme oxygenase-1; HPH, hypoxic pulmonary hypertension; I/R, ischemia and reperfusion; IL-6, interleukin-6; IL 8, interleukin 8; MAPK, mitogen-activated protein kinase; MIRI, myocardial ischemia/reperfusion injury; MMP-9, metalloproteinases-9; NaHS, sodium hydrosulfide; NO, nitric oxide; NRW, no reported weight; SNAP, S-nitroso-N-acetylpenicillamine; SNO, protein S-nitrosylation; SSH, protein S-sulfhydration; TNF-α, tumor necrosis factor-alpha; VSMCs, vascular smooth muscle cells

**Table 3:** Overview of experimental studies on sulfur dioxide induced protection

Sulfur dioxide					
Model	End point	Treatment	Targets	Results	Refs.
In vivo					
Male Wistar rats, weighing 200–250 g	Evaluation of effects of SO_2_ on heart performance and cardiomyocyte apoptosis after ISO injury	ISO + SO_2_ donor (NaHSO_3_ and Na_2_SO_3_, 1:3 MM ratio)	Cardiomyocyte apoptosis, Bcl-2, Bax, Cyt c, caspase-9/3, mPTP, mitochondrial membrane potential	Improved heart performance, relieved myocardial injury, reduced apoptosis, up-regulated bcl-2, down-regulated bax, stimulated mitochondrial potential, closed mPTP, reduced Cyt c release, decreased caspase-9/3 activities	[84]
Male Wistar rats, weighing 220–250 g	Evaluation of effects of endogenous SO_2_ on heart performance and redox stress, and response to ISO for SO_2_/GOT pathway	ISO + SO_2_ donor (NaHSO_3_ and Na_2_SO_3_, 1:3 MM ratio)	Endogenous SO_2_/GOT pathway, myocardial structures, oxidative stress, antioxidative capacity	Improved heart performance. Ameliorated myocardial damage, improved pathological structure of the myocardium and mitochondria. Decreased oxidative stress, increased antioxidative capacity	[105]
Male Wistar rats, weighing 250–300 g	Protection by SO_2_ preconditioning against MIRI (infarct size)	SO_2_ donor preconditioning, PI3K inhibitor LY294002	MIRI, LDH, CK, Caspase-3, Caspase-9, p-PI3K	Reduced infarct size, decreased LDH and CK activities, Lower caspase-3 and -9 activities, increased p-Akt and p-PI3K expression	[235]
Ex vivo/in vitro					
Isolated aortic rings from Male Wistar rats, weighing 250 ± 5 g	Endogenous production of the SO_2_ in vascular tissues (aortic rings) and vascular protection	SO_2_ donor (NaHSO_3_ and Na_2_SO_3_)	Endogenous SO_2_, aspartate aminotransferase, L-type Ca^2+^ channel	Aorta had the highest SO_2_ content. SO_2_ derivatives (physiological dose) slightly relaxed artery rings, higher doses showed concentration-dependent relaxation. Nicardipine eliminated vasorelaxant response to SO_2_. Incubation with nicardipine or SO_2_ derivatives prevented vasoconstriction induced by Bay K8644 (L-type Ca^2+^ channel agonist)	[37]
Isolated hearts from Male Wistar rats, weighing 250–300 g	Evaluation of heart performance and involvement of the ERK1/2 signaling pathway after SO_2_ preconditioning	SO_2_ donor (NaHSO_3_ and Na_2_SO_3_), ERK1/2 inhibitor (PD98059)	ERK1/2 signaling pathway	I/R-induced cardiac dysfunction and increased phosphorylated-ERK1/2 protein. SO_2_ preconditioning suppressed phosphorylated-ERK1/2 and improved heart performance. PD98059 pretreatment prevented the effects of SO_2_ preconditioning	[73]

Bcl-2, B-cell lymphoma-2; Bax, Bcl-2-associated protein x; Cyt c, cytochrome c; CK, creatine kinase; ERK1/2, extracellular signal-regulated kinase1/2; GOT, glutamic oxaloacetic transaminase; ISO, isoproterenol; I/R, ischemia/reperfusion; LDH, lactate dehydrogenase; MIRI, myocardial ischemia/reperfusion injury, mPTP; mitochondrial permeability transition pore; Na_2_SO_3,_ sodium sulfite; NaHSO_3_, sodium bisulfite; p-Akt/PKB, phospho-protein kinase B; PI3K, phosphoinositide 3-kinase; p-PI3K, phospho- PI3K; SO_2_, sulfur dioxide; PD98059, mitogen-activated protein kinase/ERK pathway inhibitor

**Table 4:** Overview of experimental studies on carbon monoxide induced protection

Carbon monoxide					
Model	End point	Treatment	Targets	Results	References
In vivo					
Yorkshire White pigs (Porcine Model of AMI), weighing approximately 25 kg	Efficacy and safety of CORM-A1 to reduce infarct size and improve heart performance	CORM-A1 (4.27 mM) infusion at 15 min post-AMI	CO, infarct size, cardiac biomarkers, ejection fraction, inflammation, cell proliferation, apoptosis	CORM-A1-treated pigs showed a significant reduction in infarct area, improved heart performance, and lower myocardial injury markers. The cardioprotective effects were associated with reduced inflammation and cell proliferation. No evidence of hepatic or renal toxicity was observed	[82]
Wild-type male ICR mice, weighing 35.1 ± 1.2 g	Impact on apoptotic signaling pathways in the setting of late PC and MIRI	CORM-3 pretreatment to induce late PC; iCORM-3 (which does not release CO) as control	Apoptotic signaling pathways, transcription factors (NF-κB, STAT1/3, Nrf2), protective (HO-1, COX-2, Ec-SOD), and antiapoptotic proteins (Mcl-1, c-FLIP(S), c-FLIP(L))	CORM-3 significantly reduces markers of apoptosis (cleaved lamin A, cleaved caspase-3, cleaved PARP-1) after ischemia/reperfusion injury. Activation of NF-κB, STAT1/3, Nrf2, and subsequent upregulation of cardioprotective and antiapoptotic proteins observed, contributing to late preconditioning-mimetic infarct-sparing effects	[195]
Ex vivo/in vitro					
Freshly isolated cardiomyocytes from male wild-type C57BL/6 J mice, 8–12 weeks old (NRW)	Evaluation of CORM-3-mediated cytoprotection involving the modulation of pH regulation	CORM-3 (50 μM) at reoxygenation; iCORM-3 as control	CO, pH regulators (NHE, NBC), mitoKATP channels, MEK1/2	CORM-3 reduces mortality in cardiomyocytes during hypoxia-reoxygenation. The cardioprotective effect involves CO and modulation of pH regulation, specifically inhibiting a bicarbonate transporter (Na^+^/HCO_3_^−^ symporter). Activation of mitoKATP channels and MEK1/2 is required for this protective effect	[157]

AMI, acute myocardial infarction; CO, Carbon monoxide; CORM-3 CO-releasing molecule-3; CORM-A1, water-soluble CO releaser; COX-2, cyclooxygenase-2; c-FLIP(S) and c-FLIP(L), Cellular FLICE Inhibitory Protein; Ec-SOD, extracellular superoxide dismutase; HO-1, heme oxygenase; ICR, institute of cancer research; Mcl-1, antiapoptotic proteins in the mitochondria-mediated; MEK1/2, mitogen-activated protein kinase kinase 1/2; MIRI, myocardial ischemia/reperfusion injury; mitoKATP channels, mitochondrial ATP-sensitive K^+^; NBC, sodium/bicarbonate cotransporter; NF-κB, nuclear factor kappa-light-chain-enhancer of activated B cells, NHE, sodium–hydrogen exchanger; Nrf2, nuclear factor erythroid 2-related factor; NRW, no reported weight; PARP-1, poly (ADP-ribose) polymerase; PC, preconditioning; STAT1/3, signal transducer and activator of transcription

### Nitric oxide and cardioprotection

The importance of NO in both normal physiological processes and pathological conditions is well established. However, for a more in-depth understanding of its various roles, readers are encouraged to refer to recent reviews that offer comprehensive insights [3, 6, 7, 85, 153].

**Fig. 1:**
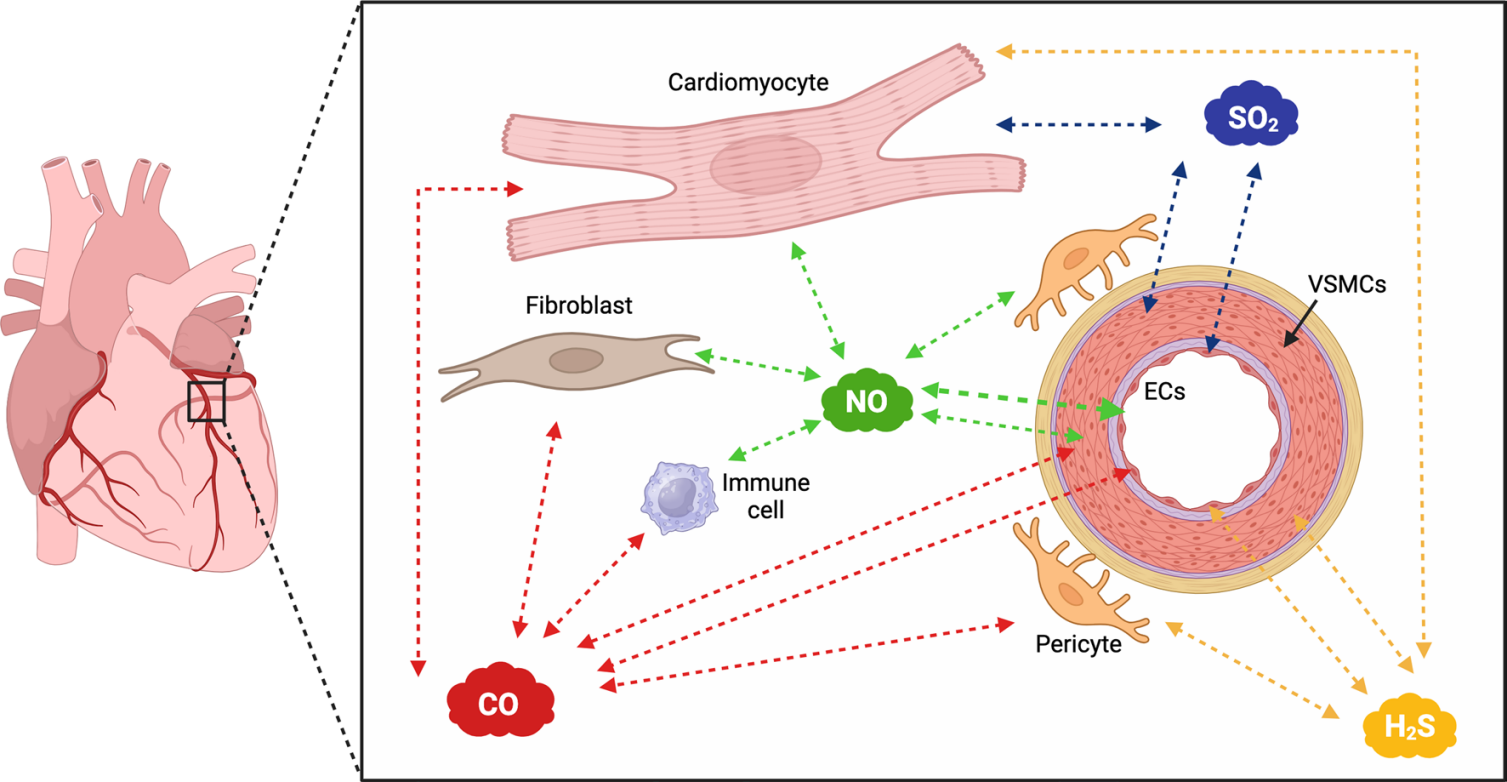
Schematic representation of endogenous gasotransmitter production and interaction on various cardiovascular cell types. In addition to the well-established role of ECs and VSMCs, immune cells and microvascular pericytes are emerging as significant contributors to cardioprotection. Green cloud: NO; yellow cloud: H_2_S; blue cloud: SO_2_; red cloud: CO. Created with BioRender.com. CO, carbon monoxide; ECs, endothelial cells; H_2_S, hydrogen sulfide; NO, nitric oxide; SO_2_, sulfur dioxide_;_ VSMCs, vascular smooth muscular cells

*Sources:* The synthesis and primary metabolic pathways of NO are extensively discussed elsewhere [9, 26]. In brief, endogenous NO is produced enzymatically through the conversion of l-arginine by a specific group of enzymes known as NO synthases (NOSs). These NOS enzymes, which exist as homodimeric oxidoreductases, are expressed constitutively in various cell types. Neuronal NOS (nNOS), inducible NOS (iNOS), and endothelial NOS (eNOS) are expressed by various tissues; therefore, they are also called NOS1, NOS2, and NOS3, respectively [3]. In particular, iNOS and nNOS highlight the involvement of novel cellular actors in cardioprotection. Various cell populations, such as immune cells [4] and pericytes [39], appear to play a role in the cardioprotective capacity of NO and other gasotransmitters (Fig. 1). In addition to these cells, emerging evidence highlights the significant role played by erythrocytes in mediating NO protective effects. Erythrocytes contribute to NO bioavailability and signaling pathways, thereby exerting a notable influence on cardioprotection against MIRI. Nitric oxide can also be generated in tissues through either direct disproportionation or the reduction of nitrate and nitrite to NO under acidic and highly reduced conditions that are present in disease states like ischemia [70, 94, 117, 119, 223]. Actually, during myocardial ischemia, NO synthesis increases independently of NOS pathways, impacting outcomes via concentration-dependent mechanisms [70]. Endogenous NO favors myocardial hibernation during ischemia by reducing O_2_ consumption and preserving function, thus preventing necrosis [117].

**Fig. 2:**
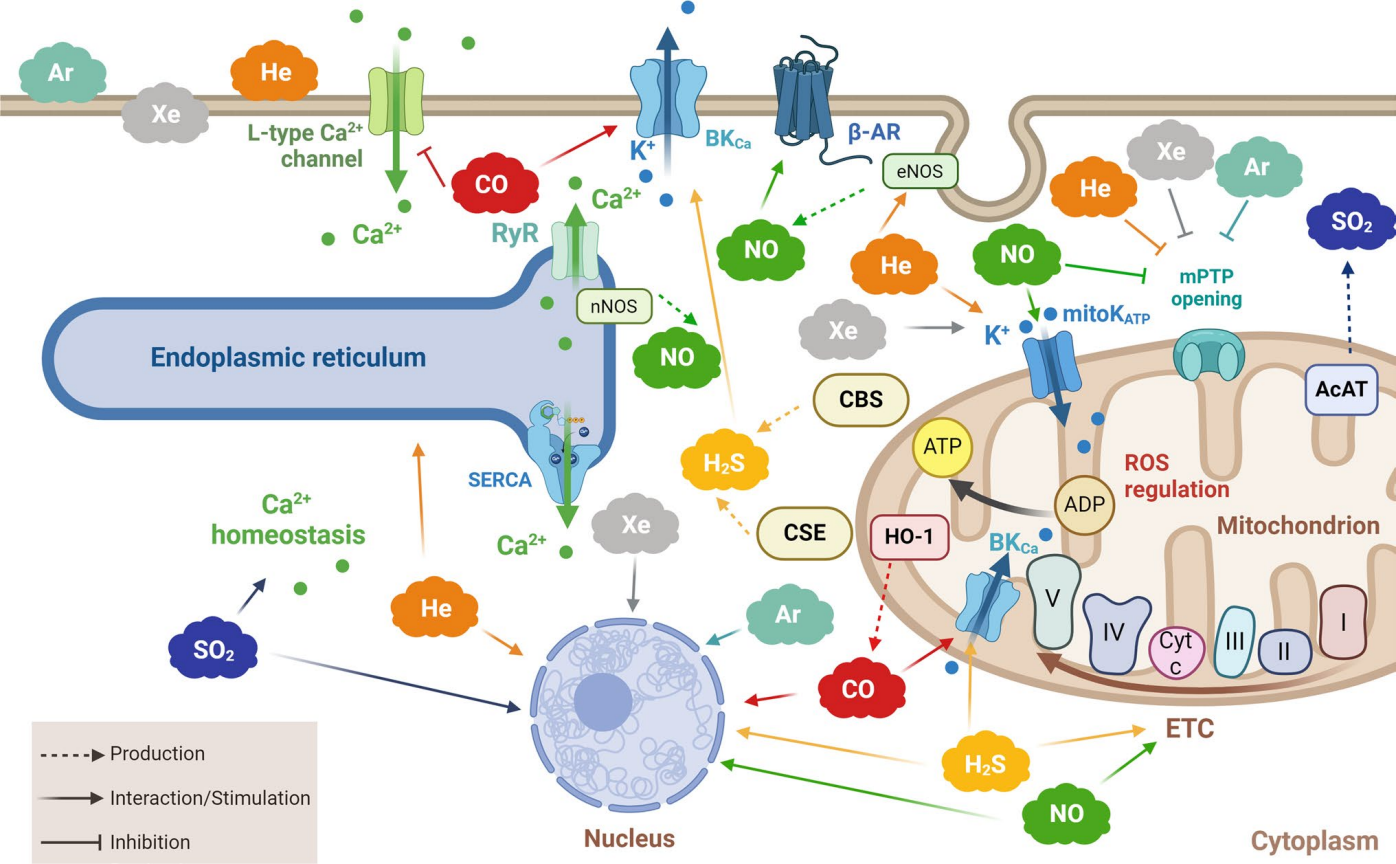
Schematic representation of noble gas and endogenous gasotransmitter interaction with cellular organelles. Gases can interact with various cellular organelles, including mitochondria, endoplasmic reticulum, and nucleus, influencing cellular signaling and function. Mitochondria play a major role (for more details on cardioprotective mechanisms of NO and H_2_S in mitochondria during ischemia/reperfusion see reference #7). Green cloud: NO; yellow cloud: H_2_S; blue cloud: SO_2_; red cloud: CO; orange cloud: He; gray cloud: Xe; light-blue cloud: Ar. Created with BioRender.com. I, II, III, IV, and V (ATP synthase) indicate respiratory chain complexes; Ar, argon; ADP, adenosine diphosphate; ATP, adenosine triphosphate; β-AR, β adrenergic receptor; BK_Ca_, large conductance Ca^2+^-activated K^+^ channels; Ca^2+^, calcium; CBS, cystine beta-synthesis enzyme; CO, carbon monoxide; CSE, cystathionine gamma-lyase; Cyt c, cytochrome c; ETC, electron transport chain; H_2_S, hydrogen sulfide; He, helium; HO-1, heme oxygenase-1; K^+^, potassium; mitoKATP, mitochondrial ATP-sensitive potassium channel; mPTP, mitochondrial permeability transition pore; nNOS, neuronal nitric oxide synthase; NO, nitric oxide; ROS, reactive oxygen species; RyR, ryanodine receptor, SERCA, sarco-endoplasmic reticulum calcium ATPase; SO_2_, sulfur dioxide; Xe, xenon

**Fig. 3:**
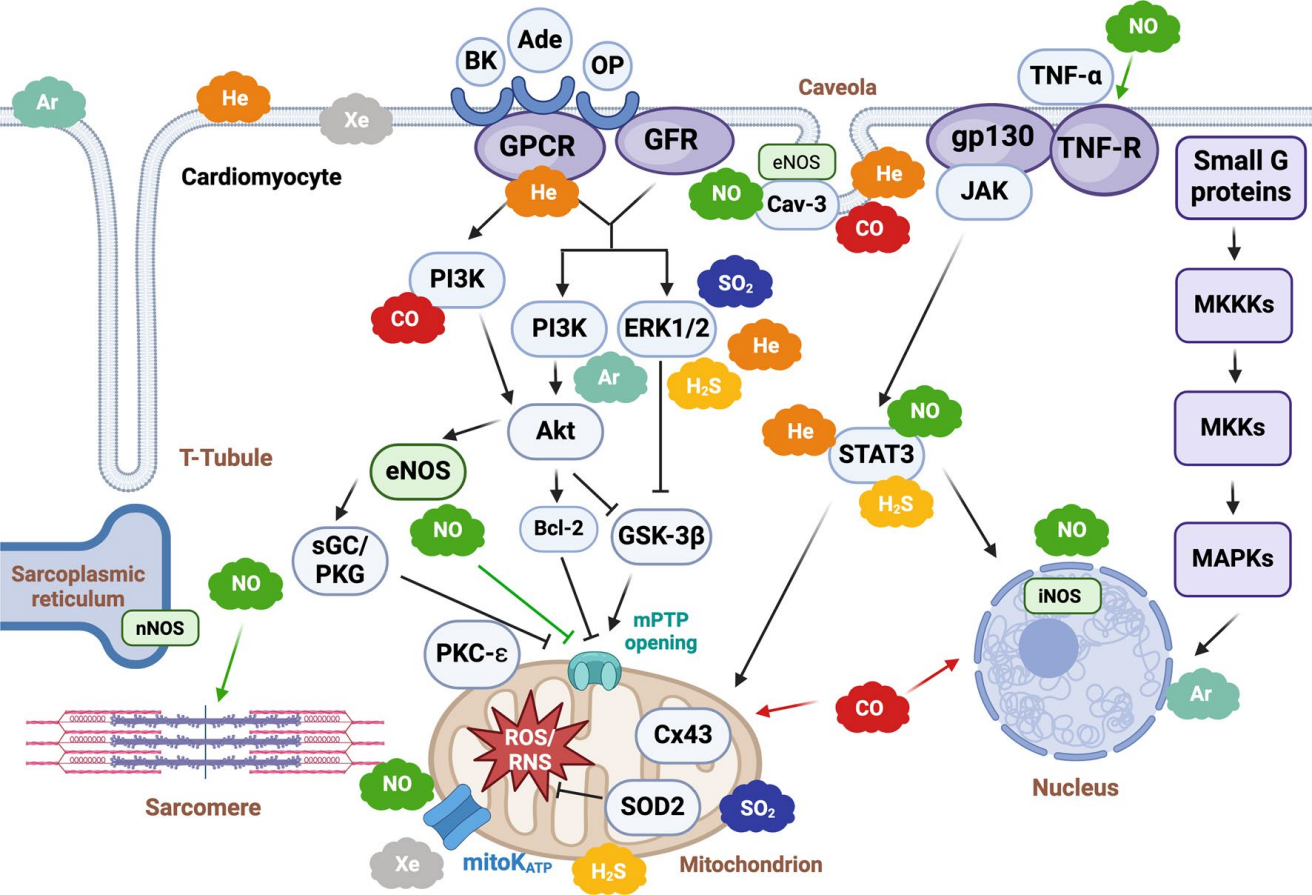
Schematic diagram illustrating the key signaling pathways of gasotransmitters and noble gases within cardiomyocytes. The diagram delineates the principal cardioprotective signaling pathways. Extracellular molecules and gases (depicted as clouds) engage with sarcolemmal receptors or function independently of receptors. This interaction triggers downstream cytosolic signaling cascades, such as the NO/PKG, RISK, and SAFE pathways, while gases also modulate or activate additional kinases not explicitly linked to the indicated pathways. Intracellularly, these pathways converge on the mitochondria, inhibiting the opening of the mPTP. Gases may also directly affect several mitochondrial components (see also Fig. 2 and reference #7). Late preconditioning involves the nucleus and transcription of enzymes such as iNOS. Gases may also modulate inotropy. Green cloud: NO; yellow cloud: H_2_S; blue cloud: SO_2_; red cloud: CO; orange cloud: He; gray cloud: Xe; light-blue cloud: Ar. Created with BioRender.com. Ade, adenosine; ADP, adenosine diphosphate; Akt/PKB, protein kinase B; Ar, argon; ATP, adenosine triphosphate; Bcl-2, B-cell leukemia/lymphoma-2; BK, bradykinin; Cav-3, caveolin-3; CBS, cystine beta-synthesis enzyme; CO, carbon monoxide; CSE, cystathionine gamma- Lyase; eNOS, endothelial nitric oxide synthase; ERK1/2, extracellular signal-regulated kinase 1/2; GFR, growth factor receptor; gp130, glycoprotein 130; GPCR, G-protein coupled receptor; GSK-3β, glycogen synthase kinase-3beta; H_2_S, hydrogen sulfide; He, helium; iNOS, inducible nitric oxide synthase; MAPK, mitogen-activated protein kinase; mitoKATP, mitochondrial ATP-sensitive potassium channel; MKKKs, MAPK kinase kinases; MKKs, MAPK kinases; mPTP, mitochondrial permeability transition pore; mTOR, mammalian target of rapamycin; nNOS, neuronal nitric oxide synthase; NO, nitric oxide; OP, opioids; PI3K, phosphatidylinositol-3-kinase; PKC-ε, protein kinase C epsilon; PKG, protein kinase G; RISK, reperfusion injury salvage kinase pathway; RNS, reactive nitrogen species; ROS, reactive oxygen species; SAFE, survivor activating factor enhancement pathway; sGC, soluble guanylyl cyclase; SO_2_, sulfur dioxide; SOD2, superoxide dismutase; SR, sarcoplasmic reticulum; STAT3, signal transducer and activator of transcription 3; TNF-α, tumor necrosis factor α; Xe, xenon

*Cardiac protection:* Recent progress in unraveling NO’s involvement in cardiac biology has highlighted its crucial role in defending against MIRI [6, 7, 9]. The cardioprotective effects of NO are not solely induced by “conditioning” mechanisms. For instance, upon β-adrenergic stimulation, the nNOS, which is bound to ryanodine receptors in the sarcoplasmic reticulum, modulates NO levels, facilitating calcium release and controlling inotropy [49] (Figs. 2 and 3). Therefore, NO plays a pivotal role in safeguarding the β-adrenergic-mediated heart function regulation. Moreover, NO influences the calcium release through S-nitrosylation, modulating key proteins, such as calcium channels. Inhibition of nNOS by superoxide (O_2_^(−)^) as well as by peroxynitrite (ONOO^(−)^, formed by the reaction of NO with O_2_^(−)^), hinders β-adrenergic stimulation, impacting calcium release and consequently cardiac inotropy [28, 55]. Altered NO levels and increased O_2_^(−)^ result in nNOS uncoupling, and blocked phospholamban phosphorylation. The formed ONOO^(−)^ affects cardiomyocyte action potentials, induces lipid peroxidation, and damages mitochondria. Moreover, ONOO^(−)^ influences cardiomyocyte function through the effects on sarco-endoplasmic reticulum calcium ATPase (SERCA). Indeed, elevated ONOO^(−)^ levels promote calcium sequestration via SERCA, affecting cardiomyocyte relaxation [15, 35]. Nitric oxide actively contributes to protection induced by various factors, such as physical exercise [42, 147] and the stimulation of β3-adrenergic receptors [126, 159]. In particular, nNOS exhibits cardioprotective effects during exercise by modulating O_2_ consumption. Endothelial NOS contributes to cardioprotection by inhibiting the β-adrenergic response through the regulation of the L-type calcium channel. During exercise, the increased ratio of eNOS dimer to monomer, coupled with reduced peroxynitrite levels, promotes eNOS dimerization and activation, further enhancing cardiac protection [40, 41, 124, 208]. During exercise, iNOS levels are low, while elevated levels are induced in reactive hypertrophy through various signaling mechanisms. Increased levels of NO during exercise enhance mitochondrial O_2_ consumption, regulated by angiotensin II in cardiomyocytes. Notably, cardiomyocytes control NO diffusion and compartmentalization, with heme-centered proteins such as cytoglobin and myoglobin scavenging locally released NO, thus regulating this gas diffusion within the heart [57, 58].

*Conditioning:* In simple terms, the previously mentioned cardioprotection protocols (namely PC, PostC and RC have revealed cooperative and protective signaling pathways, including the RISK, SAFE, and NO/cGMP/PKG pathways [46, 166–168, 183] (Fig. 3). These pathways involve phosphorylation and dephosphorylation mechanisms with various kinases, including NOS. Furthermore, redox-dependent protective pathways are implicated, where ROS, S-nitrosylation by NO, and NO derivatives such as nitroxyl (HNO) play integral roles [113]. Therefore, NO participates in signaling cascades by activating enzymes such as guanylyl cyclase (GC) and it functions as a key element in redox signaling, engaging in reactions with O_2_^(−)^ and sulfur groups.

The NO molecule plays a central role in initiating and mediating the late phase or second window of protection in ischemic PC. The cardioprotective effects of NO are particularly intriguing for the iNOS, which also displays a role in protecting the mitochondria. Actually, iNOS is primarily involved in inflammatory responses and reactive hypertrophy in the heart, but it is also involved in the second window of ischemic PC [142]. This has been demonstrated in both ischemic PC and pharmacological cardioprotection [85, 142, 195]. The widely accepted idea that increased iNOS activity in the later phase of PC enhances NO availability reinforces iNOS as a protective protein against MIRI. Yet, excessive NO production and iNOS expression and uncoupling were observed in cardiac allograft rejection, suggesting involvement of NO and O_2_^(−)^ in tissue injury through ONOO^(−)^ formation [2]. Despite limitations in current approaches, studies indicate that NO scavengers may extend acute cardiac graft survival by limiting NO’s actions and treatment with NOS inhibitors improved cardiac injury significantly [156]. However, the precise role of NO and NO/O_2_^(−)^ ratio in organ rejection is still debated, necessitating further research on their effects on different cell types.

It has been suggested that NO interacts with elements of the electron transport chain (ETC) and/or the mPTP to alleviate post-ischemic myocardial damage (Fig. 2). However, the precise molecular events of this action remain elusive. Nevertheless, this interaction with mitochondria offers a fundamental molecular explanation for the mechanism behind NO-mediated cardioprotection, emphasizing NO as a common mediator of protection across various interventions against myocardial ischemia and reperfusion [6, 7, 153]. A comparable protective function is attributed to PostC, with both protection (PC and PostC) being hindered by the NOS inhibitors [81, 108, 151].

As seen above and summarized in Fig. 3 and Table 1, within the heart, NO plays a pivotal role in various signaling pathways involved in cardioprotection against MIRI. However, there is contradictory evidence that *endogenous* NO is involved in ischemic PC. For instance, endogenous NO did not alter infarct size development and was not implicated in the protection against infarction through classical ischemic PC in rabbits ex vivo [123] and pigs in vivo [158]. These findings underscore the complex and context-dependent nature of NO's cardioprotective mechanisms. It is also useful to differentiate between the NO that exerts cardioprotective effects within the heart and the NO that circulates in the form of cardioprotective, bioactive plasmatic nitrite. Circulating nitrite deriving from remote site contributes to cardioprotection by ischemic RC [166–168, 202]. These studies evidenced that circulating nitrite serves as a significant source of NO with cardioprotective properties. Furthermore, as highlighted erythrocytes contribute to the bioavailability of NO through the reduction of nitrite [51, 52, 201]. These distinct sources and mechanisms underscore the multifaceted nature of NO-mediated cardioprotection.

*Pharmacological conditioning*: Notably, drugs such as angiotensin-converting enzyme (ACE) inhibitors, statins, and angiotensin-receptor blockers, which boost NO levels, show benefits in mouse models of myocardial infarction (MI), supporting the potential importance of NO in conditioning cardioprotection [46, 100, 149, 150, 179]. Undoubtedly, the endothelium serves as the primary source of NO, yet its role in MIRI has often been overlooked, although the coronary circulation and endothelial dysfunction are implicated in MIRI as both causes and targets [65, 66]. In light of this, the replacement of endogenous NO with NO donors holds great promise in these conditions. Preclinical studies have unveiled various mechanisms of coronary microvascular injury that can be addressed through ischemic and pharmacological conditioning, suggesting potential clinical translation to enhance patient outcomes in MI [for reviews see 65–69]. When NO or HNO are administered before ischemia trigger protective responses similar to ischemic PC [113, 143, 198, 207]. For instance, the administration of exogenous NO, such as S-nitroso-N-acetylpenicillamine, demonstrated a beneficial effect in reducing infarct size [123]. In addition, the diethylamine-NO donor has been widely used as preconditioning mimetic [113, 143]. Moreover, the donors of HNO, a sibling of NO, are gaining recognition for their pharmacological attributes, which encompass offering functional assistance to failing hearts. In addition, HNO demonstrates the ability to precondition myocardial tissue, safeguarding it from MIRI, and exhibits actions that counteract vascular proliferation [113, 143, 200, 220].

To sum up, NO and derivatives have a plethora of targets, but the protective impact of NO and HNO operate mainly through mitochondria, interacting with different components of the ETC, mitochondrial ATP-sensitive K^+^ (mitoKATP) channels, and elements of the mPTP [78, 104, 150]. These interactions significantly reduce MIRI (Table 1).

### Hydrogen sulfide and cardioprotection

In addition to NO, H_2_S is regarded as a cardioprotective gaseous mediator.

*Sources:* At least, three enzymes in mammalian tissues produce H_2_S endogenously from cysteine. Actually, endogenous H_2_S is generated through enzymatic or nonenzymatic routes within mammalian tissues [14, 18, 145, 221]. Key enzymes involved in this process include cystathionine γ-lyase (CSE) and cystathionine β-synthase (CBS), both utilizing homocysteine as a substrate. In addition, 3-mercaptopyruvate sulfurtransferase (3-MST) can catalyze H_2_S synthesis in conjunction with cysteine aminotransferase (CAT). CSE exhibits localization in the kidney, liver, vessels, and heart, CBS is found in the central nervous system, and 3-MST is expressed in the liver, heart, brain and kidney. The catabolic pathways of H_2_S involve: (a) oxidation to thiosulfate catalyzed by mitochondrial thioquinone oxidoreductase, S-dioxygenase, and S-transferase; (b) generation of methyl mercaptan and dimethyl sulfide through a reaction catalyzed by cytoplasmic thiol S-methyltransferase; (c) interaction with methemoglobin leading to the production of thiol hemoglobin [7, 10, 106, 168].

*Cardioprotection:* Apart from ischemia/reperfusion injury, heart failure, hypertrophy, fibrosis, myocardial infarction, arrhythmia, and several physiological and pathological processes have all been shown to be prevented by H_2_S. Its cardioprotective effect may be attributed to mechanisms such as NO interaction, ion channel regulation, antioxidative action, mitochondrial function preservation, apoptosis reduction, anti-inflammatory responses, and angiogenic actions [6, 7, 9, 20, 114, 170]. Despite identifying multiple mechanisms, additional research is necessary to determine the precise molecular mechanism of cardioprotection in various cardiac diseases to open the door to novel therapeutic targets based on H_2_S production and/or modulation.

*Conditioning:* During MIRI, the plasma level of H_2_S and CSE in the myocardium are reduced, while the mRNA expression level of CSE is increased following reperfusion, which contributes to a sort of positive feedback. Indeed, in KO model for CSE oxidative stress and MIRI are exacerbated [89]. In rodents, conditioning protection is mainly mediated by the RISK and SAFE pathways (Fig. 3), activated at the onset of reperfusion [87, 89, 118]. Findings indicate that naturally elevated levels of H_2_S act to safeguard the heart against MIRI, suggesting their potential as significant therapeutic targets.

*Pharmacological conditioning:* The majority of studies on this gas considered exogenous donors (Table 2). The administration of different H_2_S donors before reperfusion reduces infarct size and the plasma level of troponin-I significantly in MIRI mice [89]. Pretreatment with H_2_S, acting as a modulator of an ERK1/2-dependent pathway, also reduces endothelial cell death in vitro [235]. The protective effect of H_2_S is reported when the administration is either before (PC-like) or after (PostC-like) prolonged ischemia [5–7, 9].

In a porcine model, H_2_S treatment significantly improved hemodynamics, reduced infarct size, and displayed anti-inflammatory effects, suggesting potential therapeutic utility in clinical settings encountering MIRI [189].

Besides the influence on RISK and SAFE pathways, the cardioprotective effects induced by exogenous sodium hydrosulfide (NaHS) depend on mitochondrial ETC enzymes and also lead to mitoKATP channel opening (Figs. 2 and 3) [6, 7, 9]. Accumulating evidence has reported a crosstalk between H_2_S and NO. In fact, CSE knockout mice displayed a reduction in NO levels due to reduced eNOS expression. In this model, the treatment with H_2_S induces an increase in NO bioavailability and restores eNOS protein expression, which subsequently attenuated oxidative stress and MIRI. Recently, it was observed that S-sulfhydration, a post-translational modification involving the interaction of H_2_S with cysteine residues in proteins, alters the structure and biological activities of protein targets. In mice, application of pharmacological postconditioning using NaHS at the onset of reperfusion significantly enhanced S-nitrosylation of cardioprotective proteins, concurrently mitigating post-ischemic contractile dysfunction and reducing infarct size [198].

In the apolipoprotein E knockout model, the administration of NaHS induces increased plaque stability and blood lipid levels and reduced plaque formation [222]. Intriguingly, the Andreadou group displayed an interplay between eNOS, H_2_S, and CO-synthesizing enzymes in human atheroma in plaque stability and simvastatin effects [182]. Limited research has explored the interplay between CO and H_2_S in signal transduction, and the synergistic mechanism remains incompletely elucidated (see also “Carbon monoxide and cardioprotection”).

### Sulfur dioxide and cardioprotection

Sulfur dioxide stands out as an additional gasotransmitters, showcasing diverse biological impacts, including antioxidative, anti-inflammatory, antihypertensive, and antiatherogenic effects.

*Sources:* Pioneering discoveries reveal that endogenous SO_2_ formation occurs within the cardiovascular system, exerting a prominent vasorelaxant influence [37, 74]. Subsequent findings identified SO_2_ products in various organs, such as the brain, stomach, lungs, liver, spleen, and heart [75, 109]. Aspartate aminotransferase is indicated as the key enzyme for SO_2_ synthesis. Conversely, SO_2_ catabolism involves the hydrogenation of bisulfite and sulfite ions, which are then oxidized to sulfate [74, 75].

*Cardioprotection:* Current knowledge establishes SO_2_’s engagement in apoptosis and oxidative stress, elevating antioxidant enzyme expression (e.g., SOD2 and GSH-Px1) and decreasing ROS production [105]. Simultaneously, in rats with isopropylarterenol-induced myocardial injury the antiapoptotic effects of SO_2_ are attributed to increased B-cell leukemia/lymphoma-2 (Bcl-2) expression, Bcl-2 associated protein x inhibition, mitochondrial membrane stabilization, decreased cytochrome c release, and diminished caspase activation [84]. Furthermore, SO_2_ plays a role in intracellular calcium homeostasis, with disruptions linked to cell death in numerous pathological conditions [74, 75]. Sulfur dioxide emerges as a pivotal regulator in various biological processes under both normal and pathological conditions associated with cardiovascular diseases. Recent investigations into the impact of SO_2_ on cell apoptosis have gained significant attention, revealing its regulatory influence on vascular smooth muscle cells, endothelial cells, cardiomyocytes, and other cells implicated in the pathogenesis of arterial hypertension and myocardial damage [234]. This multifaceted role positions SO_2_ as a crucial player in cardiovascular health, influencing cellular processes and offering potential therapeutic avenues in cardiac disorders.

*Ischemic and pharmacological conditioning:* Preconditioning with SO_2_ diminished myocardial infarct size, plasma lactate dehydrogenase, and creatine kinase activities in rats, alongside reducing myocardial caspase-3 and -9 activities. In addition, this pretreatment substantially elevated the expression levels of myocardial phosphorylated‑protein kinase B (p-PKB/p‑AKT) and phosphorylated‑PI3K-p85. Notably, the administration of the PI3K inhibitor LY294002 effectively nullified all the beneficial effects initiated by SO_2_ preconditioning. Furthermore, exogenous SO_2_ preconditioning improved cardiac function and reduced phosphorylated-ERK1/2 protein expression in isolated rat hearts with MIRI, suggesting the involvement of the ERK-MAPK pathway. Inhibition of ERK1/2 activation by the inhibitor PD98059 abolished the cardioprotective effects of SO_2_ [73]. Additional data on SO_2_ preconditioning strongly support the theory that the PI3K/Akt and ERK pathways play pivotal roles in mediating the protective effects against MIRI in rats [234] (Table 3; Fig. 3).

### Carbon monoxide and cardioprotection

*Sources:* Carbon monoxide, being an easily diffusible gaseous molecule, originates from diverse sources within biological systems. The primary origin in mammals is attributed to heme oxygenase (HO) and HO-like activity. Carbon monoxide is generated through the degradation of the heme group via HO, which includes the constitutive forms HO-2 and HO-3, and the inducible form HO-1 [6, 19, 22, 53].

*Cardioprotection:* In the cardiovascular system, including the heart, CO-induced vasodilation occurs through the classical NO pathway, involving soluble GC (sGC)/cGMP, and PKG pathways. In addition, CO impedes endothelin-1 synthesis, prevents platelet aggregation, and inhibits L-type Ca^2+^ channels, collectively contributing to the cytoprotection of cardiomyocytes [22, 171]. Upregulation of HO-1 and its metabolites is known to activate various cell protection pathways, particularly during MI and hypoxia. Notably, during hypoxia and MI, there is a substantial increase in HO-1 expression, leading to heightened CO production [53]. Indeed, HO-1, in part, contributes to damage recovery and tissue repair by generating bioactive products such as CO. Carbon monoxide initiates a cardioprotective and antiapoptotic environment in the myocardium, akin to the effects observed during the late phase of preconditioning. This suggests that the presence of CO induces a state in the myocardium that mimics the delayed preconditioning, contributing to protection against cardiac damage and apoptosis [195].

Pathophysiological interactions between CO and H_2_S have been validated in a model of myocardial ischemic PC. In a rat model of pulmonary hypertension, H_2_S was observed to elevate HO-1 expression in the pulmonary artery and increase CO concentration in plasma [20, 164]. Carbon monoxide demonstrates cardioprotective properties, primarily through the opening of mitoKATP channels. This process inhibits the long-lasting opening of the mPTP and regulates mitochondrial ROS production. Although information on the cardioprotective effects of CO is limited, it is suggested that at low concentrations, CO facilitates the preservation of mitochondrial function. Under these conditions, CO has been observed to maintain the stable potential of the mitochondrial membrane, particularly during the ischemia/reperfusion period [9, 131, 171]. The mitochondrial level is a key site of CO action, where it induces the opening of mitoKATP channels and inhibits mPTP opening [22, 171]. Moreover, CO modulates several signal pathways implicated in cardioprotection, including NO/GC, MAPK, and ROS action (Fig. 3). In addition, CO has the capacity to impede platelet activation through both cGMP-dependent and cGMP-independent pathways [171]. In addition to the NO and cGMP pathways, CO exerts its anti-inflammatory properties by inhibiting cytokines such as tumor necrosis factor-alpha (TNF-α) and interleukin-1β (IL-1β) [135]. Apart from its anti-inflammatory effects, CO has proven its value in the field of organ transplantation, leading to improved outcomes [132]. Furthermore, in a porcine model of cardiopulmonary bypass, CO demonstrated its beneficial effects by enhancing cardiac energetics [98].

HO-1 expression and activity increase in hypoxic states, decrease during reperfusion, and correlate with heightened myocardial damage [171]. Protective action of CO is attributed to increased heme availability, a substrate for HO-1, and elevated HO-1 activity during reperfusion [22]. Indeed, the absence of HO-1 significantly increases vulnerability to ischemic injury [79].

*Pharmacological conditioning:* The majority of studies deal with exogenous CO, whose administration simulates HO-1 induction, resulting in heightened inotropy, reduced apoptosis, and diminished infarct size in rat hearts. Moreover, CO operates through a PKB/Akt-GSK-3β-nuclear factor erythroid 2-related factor (Nrf2) pathway, fostering mitochondrial biogenesis. Indeed, the influence of HO-1/CO on mitochondria is pivotal for the differentiation of stem cells into cardiac myocytes within the heart [155, 197]. Mitochondrial function likely plays a crucial role in CO-induced cardioprotection, impacting gene regulation, downstream signaling, and cell survival. Protective role of CO in the heart relies on HO-1 levels, triggered by tissue injury leading to the release of cellular contents, including elevated heme levels. This, subsequently, initiates HO-1 and endogenous CO generation, both highly cardioprotective and replicable by exogenous CO. Subtle concentrations of CO form transient bonds with cytochrome c oxidase, generating minimal levels of ROS. These ROS, in turn, activate signaling complexes involving Nrf2, hypoxia-inducible factor 1alpha (HIF-1α), and nuclear factor-kappa B (NF-κB)/inhibitor of NF-κB. Carbon monoxide also boosts the expression of peroxisome proliferator-activated receptor-gamma coactivator-1α, Nrf1, and Nrf2, thereby promoting CO-induced mitochondrial biogenesis for tissue preservation and regeneration [16, 21, 155].

Preconditioning triggered by CO demonstrates its ability to offer neuronal protection against apoptosis. This phenomenon highlights the capacity of CO to create a preconditioned state that safeguards cells from the process of programmed cell death [165, 206]. Two recent studies highlight the therapeutic potential of CO-releasing molecules (CORMs) in myocardial protection. Portal et al. show that CORM-3, a water-soluble CO-releasing molecule, exhibits substantial cardioprotective effects against hypoxia-reoxygenation in adult cardiomyocytes, emphasizing its potential as a therapeutic agent for MI [157]. In another study, Iqbal et al. demonstrate that CORM-A1 significantly reduce infarct size in a porcine model of acute MI [82] (Table 4).

## Noble gases

Noble gases belong to a family of six naturally occurring gases: helium (He), neon (Ne), argon (Ar), krypton (Kr), xenon (Xe) and radioactive radon (Rn) [72, 209, 231].

Noble gases possess a defining trait: a fully occupied outer shell of valence electrons. This characteristic imparts inertness or, at the very least, diminished reactivity with other compounds under typical atmospheric pressure and temperature conditions. It is this distinctive feature that leads to their common designation as *inert gases*.

Nonetheless, a few of these noble gases exhibit potent biological effects. For instance, Xe is the only noble gas that shows anesthetic properties under normobaric conditions, while none of the other five gases has these properties [27, 72].

There is increasing evidence during the last 20 years that these so-called inert gases have strong cardioprotective and neuroprotective qualities [32, 50, 72, 116, 183, 209, 213, 214].

However, how these gases exert their cardioprotection is still a matter of debate. For Xe, it was shown that it may cause conformational changes in two proteins: annexin V, which has a hydrophilic pore inside and is meant to bind to cell membranes via a calcium-dependent mechanism, and urate oxidase, an intracellular globular protein with large hydrophobic cavities [25]. The Xe binding sites of the corresponding proteins consist of flexible gas cavities devoid of water. It has been demonstrated that Xe affects a number of cell membrane receptors. These include the nicotinic acetylcholine receptor [223], the 5-hydroxytryptamine 3 receptor [199], the plasmalemmal adenosine triphosphate-sensitive potassium channels (KATP) channel [11], the two-pore domain potassium channel TREK-1 [54], and the N-methyl-D-aspartate (NMDA) receptors [47]. In particular, it has been shown that at the glycine site of the NMDA receptor, Xe competes with the co-agonist glycine [33, 59]. However, this knowledge is primarily derived from neural cells. Thus, it is unclear if these Xe effects on neural cells also contribute to its myocardial protection. Nonetheless, it has been shown that regarding cardioprotection KATP channels—at least the mitoKATP channels—play a crucial role in the PC of the heart [134] and, therefore, might be a key mediator in noble gas-induced cardioprotection.

Here, we will briefly report and discuss the recent studies displaying cardioprotective effects of some noble gases (Ar, Xe, and He) with the main focus on the underlying molecular mechanism involved. A summary of the experimental studies on noble gas-induced cardioprotection can be found in Tables 5, 6 and 7 and Figs. 2 and 3.

**Table 5:** Overview of experimental studies on argon-induced protection

Argon					
Model	End point	Treatment	Targets	Results	Refs.
In vivo					
Male New Zealand white rabbits, weighing 2.5–3.0 kg	Evaluation of infarct size limiting effects by noble gases and role of PI3K, ERK1/2, and p70S6K, and mPTP	30-min LAD coronary artery occlusion and 3-h reperfusion, 3 × 5 min 70% He–, Ne–, or Ar–30% O_2_, interspersed with 5 min of 70% N_2_–30% O_2_ before LAD occlusion	Infarct size, prosurvival signaling kinases and mPTP opening	Reduction of myocardial infarct size, activation of prosurvival signaling kinases and inhibition of mPTP opening	[141]
Male Sprague–Dawley rats, 12–15 weeks old (NRW)	Evaluation of argon PC effects on recovery of heart performance following cardioplegic arrest	3 × 5 min of Ar (50% argon, 21% O_2_ and 29% nitrogen) interspersed with 3 × 5 min (79% nitrogen and 21% O_2_)	levels of myocardial phosphocreatine, ATP levels, JNK, and HMGB1	Enhanced recovery of cardiac output, stroke volume, external heart work and coronary flow. Maintained ATP levels, reduction in activation of JNK and expression of HMGB1 protein	[90]
Male Wistar rats, weighing 240–380 g	Evaluation of Ar protective effects on recovery of heart performance in a PostC protocol	30-min ischemia receiving a mixture of Ar (80%) and O_2_ (20%) for 20 min, starting 5 min before reperfusion (instead of 80% N_2_ + 20% O_2_ in control)	RISK pathway (PI3K/Akt and ERK1/2)	Reduction of contractile dysfunction and involvement of the reperfusion injury salvage kinase pathway	[99]
Ex vivo/in vitro					
Human cardiac myocyte-like progenitor cells from patients undergoing heart transplantation	Evaluation of Ar effects in human model of OGD	OGD and reperfusion	Akt, ERK1/2, p38 MAPkinase and JNK, apoptosis	Reduction apoptosis via Akt, ERK1/2, and JNK	[163]
Primary isolated cardiomyocytes from neonatal rats (NRW)	Effect of Ar PC in cardiomyocytes within the first and second window of PC	50% Ar for 1 h, followed by hypoxia (< 1% O_2_) for 5 h either within the first (0–3 h) or second window (24–48 h) of preconditioning	HSP27, SOD2, VEGF and iNOS	Increase of cell viability, increased mRNA expression of HSPB1, HSP27, SOD2, VEGF and iNOS	[114]

Akt/PKB, protein kinase B; Ar, argon; ATP, adenosine triphosphate; ERK1/2, extracellular signal-regulated kinase; He, helium; HMGB1, high mobility group box 1; HSPB1, heat-shock protein beta-1; HSP27, heat-shock protein 27; iNOS, inducible nitric oxide synthase; LAD, left anterior descending coronary artery; mPTP, mitochondrial permeability transition pore; Ne, neon; OGD, oxygen–glucose deprivation; p38 MAPkinase, p38 mitogen-activated protein kinases; p70S6K, 70-kDa ribosomal protein s6 kinase; PI3K, phosphoinositide 3-kinases; JNK, c-JUN-n terminal; NRW, no reported weight; O_2_, oxygen: PC, preconditioning, PostC, postconditioning; RISK, reperfusion injury salvage kinase; SOD2, superoxide dismutase 2; VEGF, vascular endothelial growth factor

**Table 6:** Overview of experimental studies on xenon induced protection

Xenon					
Model	End point	Treatment	Targets	Results	Refs.
In vivo					
German land-race pigs, weighing 34.9 ± 2.3 kg	Evaluation of the influence of isoflurane or Xe on right ventricle infarct size and inflammation	90-min ligation of the distal right coronary artery, 120-min reperfusion. 0.55 MAC isoflurane or xenon starting 60 min before ischemia	Infarct size, infiltration of neutrophils; MPO activity and plasma concentrations of TNF-α and IL-6	Reduction of myocardial infarct size and inflammation	[62]
Male New Zealand white rabbits, weighing 2.5–3.0 kg	Evaluation of the influence of Xe on infarct size, RISK and mPTP opening	30-min LAD occlusion and 3-h reperfusion, 3 × 5 min 70% He–, Ne–, or Ar–30% O_2_, interspersed with 5 min of 70% N_2_–30% O_2_ before LAD occlusion	Prosurvival signaling kinases and mPTP opening	Reduction of myocardial infarct size and mPTP opening	[141]
Male Wistar rats, weighing 280–340 g	Evaluation of the influence of Xe-PC on infarct size, RISK and mPTP opening	30-min LAD occlusion and 2-h reperfusion. 3 × 5-min cycles of 70% Xe/30% O_2_ interspersed with O_2_/N_2_ mixture followed by a 15-min memory period	Akt, and GSK-3β, mitochondrial O_2_ consumption and mPTP opening	Reduction of myocardial infarct size, preserved heart performance, inhibited Ca^2+^-induced mPTP opening	[120]
Male Wistar rats, weighing 200–250 g	Evaluation of the influence of Xe-LPC on infarct size and role of COX-2	xenon induces 24-h late myocardial preconditioning	Infarct size, COX-2 expression	Reduction of myocardial infarct size, involvement of COX2 in Xe-LPC	[211]
Male Wistar rats, weighing 300–450 g	Evaluation of the influence of Xe-PC on infarct size, and PKC-ε, p38 MAPK	25 min of LAD, 120 min of reperfusion. 3 × 5-min Xe or Isoflurane interspersed with two 5 min and one final 10-min washout period. Positive control IPC during three 5 min	Infarct size, PKC-ε, p38 MAPK	Reduction of myocardial infarct, activation of PKC-ε and its downstream target p38 MAPK	[218]
Male Wistar rats, weighing 200–250 g	Evaluation of the influence of Xe-PC on infarct size, and various protective pathways	25 min of LAD, 120 min of reperfusion 3 × 5-min Xe (or Isoflurane) interspersed with two 5-min and one final 10-min washout period	Infarct size, MAPKAPK-2, HSP27, PKC and p38 MAPK, F-actin mitoKATP channels and PDK-1; ERK1/2 and JNK	Reduction of infarct size; translocation of HSP27 to the particulate fraction and increased F-actin polymerization. F-actin and pHSP27 were colocalized after Xe-PC. Activation of PKC-ε and of PDK-1, and mitoKATP channels activation of ERK but not JNK in Xe-PC	[216, 217, 219]
Ex vivo/in vitro					
Isolated heart, from immature (2–3 weeks old) New Zealand rabbit hearts (NRW)	Evaluation of the influence of Xe-PC on heart performance	4 × 5-min 75% Xe + 25% O_2_ in the Langendorff model, 60-min ischemia and 180-min reperfusion	Heart performance, mitoKATP channel	Improved heart performance, Xe prevents opening of mitoKATP channels	[101]
Primary isolated cardiomyocytes from hearts of postnatal Wistar rats, 2–4 days old (NRW)	Evaluation of the influence of isoflurane, levosimendan or Xe-LPC on cell vitality and several distinct pathways	H/R 24 h prior to hypoxia, cells pre-treated with or without metoprolol (β-blocker) and preconditioned with isoflurane, levosimendan or Xe	COX-2, HIF-1α, VEGF, iNOS/eNOS	Elevated content of VEGF and HIF-1α by Xe pretreatment. Significant elevation of mRNA expression of iNOS by Xe	[50]

Akt/PKB, protein kinase B; Ar, argon; COX-2, cyclooxygenase-2; ERK1/2, extracellular signal-regulated kinase; GSK-3β, glycogen synthase kinase 3β;HIF-1α, hypoxia-inducible factor 1α; H/R, hypoxia/reoxygenation; He, helium; HSP27, heat-shock proteins 27; pHSP27, phosphorylated HSP27; IL-6, interleukin-6; IPC, ischemic preconditioning; LAD, left anterior descending; LPC, late preconditioning; MAC, minimum alveolar concentration; MAPKAPK-2, mitogen-activated protein kinase (MAPK)-activated protein kinase-2; mitoKATP channels, mitochondrial KATP channel; MPO, myeloperoxidase; mPTP, mitochondrial permeability transition pore; N_2_, nitrogen; Ne, neon; NOS, nitric oxide synthase; iNOS, inducible NOS; eNOS, endothelial NOS; NRW, no reported weight; O_2_, oxygen; PC, preconditioning; PDK-1, pyruvate dehydrogenase kinase-1; P70S6K, 70-kDa ribosomal protein s6 kinase; PKC-ε, protein kinase C-ε; p38 MAPK, p38 mitogen-activated protein kinase; RISK, reperfusion injury salvage kinases; TNF-α, tumor necrosis factor-α; VEGF, vascular endothelial growth factor; Xe, xenon; JNK, c-Jun N-terminal kinase

**Table 7:** Overview of experimental studies on helium-induced protection

Helium					
Model	End point	Treatment	Targets	Results	Refs.
In vivo					
Male New Zealand white rabbits, weighing 2.5- 3.0 kg	Evaluation of He-PC and transient alkalosis during early reperfusion on infarct size and mPTP	3 cycles of 70% He-30% O_2_ before LAD occlusion with/without transient alkalosis (pH = 7.5) before reperfusion	Infarct size, pH	Transient alkalosis during early reperfusion abolished He-PC	[136]
Male New Zealand white rabbits, weighing 2.5- 3.0 kg	Evaluation of He-PC on infarct size and role of opioid receptors	1 or 3 cycles of 70% He-30% O_2_, morphine (0.1 mg/kg intravenously), or the nonselective opioid antagonist naloxone (6 mg/kg intravenously) before LAD occlusion	Infarct size, opioid receptor	Opioid receptors mediated He-PC and its augmentation by morphine in vivo	[137]
Male New Zealand white rabbits, weighing 2.5- 3.0 kg	Evaluation of He-PC on infarct size and role of ROS and mito KATP channels	1 or 3 cycles of 70% He-30% O_2_, with/without the NAC, 2-MPG, 5-HD before LAD occlusion	Infarct size, ROS, mitoKATP	ROS and mitoKATP channels mediate He-PC in vivo	[138]
Male New Zealand white rabbits, weighing 2.5- 3.0 kg	Evaluation of He-PC on infarct size and role of NO in helium-induced cardioprotection	1 or 3 cycles of 70% He-30% O_2_, with/without the L-NAME, AG, 7-NI before LAD occlusion	Infarct size, NO	Cardioprotection by He was mediated by NO that was probably generated by eNOS in vivo	[139]
Male New Zealand white rabbits, weighing 2.5–3.0 kg	Evaluation of He-PC on infarct size and role of GSK or p53 on mPTP	30-min LAD occlusion and 3-h reperfusion. 3 × 5-min He 70%, SB21, PIF	infarct size, GSK-3β or p53	Inhibition of GSK-3β or p53 by He lowers the threshold of He-PC via mPTP	[137]
Male New Zealand white rabbits, weighing 2.5–3.0 kg	Evaluation of He- PC on infarct size and role of RISK and inhibition of mPTP opening	30-min LAD occlusion and 3-h reperfusion, 3 × 5-min 70% He–, Ne–, or Ar–30% O_2_, interspersed with 5 min of 70% N_2_–30% O_2_	Prosurvival signaling kinases and mPTP opening	Reduction of myocardial infarct size, activation of prosurvival signaling kinases and inhibition of mPTP opening	[140]
Male Wistar rats, weighing 354–426 g	Evaluation of He-PostC regulation of Cav-1 and Cav-3 expression and role of RISK pathway	25-min ischemia, followed by reperfusion (5, 15 or 30 min). He-PostC I/R and 70% He ventilation during reperfusion (I/R + He 5/15/30 min)	ERK1/2 and Akt, levels of Cav-1 and Cav-3 in myocardial tissue AAR and NAAR and blood	He-PostC regulated Cav-1 and Cav-3 and activated RISK pathway kinases ERK1/2 and AKT	[45]
Male adult Wistar rats (NRW)	Evaluation of He-PostC on infarct size and regulation of immune response	15, 30, or 60 min of 70% He during reperfusion in an LAD infarct size model	Infarct size, cytokines	Only 15 min of He reduced infarct size. CINC-3 and IL-1β were found after 30 or 60 min of He inhalation	[127]
Male Wistar rats, weighing 354–426 g	Evaluation of He-PostC on genes involved in necrosis, apoptosis and autophagy	5, 15 or 30 min of He-PostC	Histology and PCR array analysis of genes involved in necrosis, apoptosis and autophagy	He-PostC caused by a switch from pro-cell-death signaling to activation of cell survival mechanisms	[128]
WKY and SHR rats, 12–14 weeks old (NRW)	Evaluation of He-PostC and addition of LPC and EPC on infarct size in hypertensive rat hearts	70% He for 15 min after index ischemia, combined with 15-min He 24 h prior to index ischemia, triple intervention with additional 3 short cycles of 5-min He inhalation shortly before ischemia	Infarct size, GSK-3β and PKC-ε	Triple intervention of He-PostC results in cardioprotection in SHR. In WKY rats, the triple intervention does not further augment protection. GSK-3β and PKC-ε are not involved	[129]
Male Wistar rats, weighing 328 ± 19 g	Evaluate of He-induced late PC on infarct size and COX-2 activity	70%, 50%, 30%, and 10% He for 15 min 24 h before ischemia/reperfusion injury	Infarct size, COX-2	He induces late preconditioning with induction of COX-2	[76]
Male ZL rats, weighing 248 ± 5 g, and male ZO rats, weighing 334 ± 5 g	Evaluation of He-PC and He-PostC on infarct size, RISK and mPTP	25 min of ischemia, 120-min reperfusion. 3 × 5-min or 6 × 5-min 70% He, postconditioning groups 70% He for 15 min at the onset of reperfusion in ZL and ZO rats	Infarct size, ERK1/2, Akt, GSK-3β	He-PC was abolished in ZO rats GSK-3β phosphorylation was reduced after He application in ZL but not in ZO rats	[77]
Young Wistar rats, 2–3 months old, and aged Wistar rats, 22–24 months old (NRW)	Evaluation of age-dependent He-PC on infarct size and role of K(Ca^2+^) channels	Preconditioning groups (He-PC and Age He-PC) inhaled 70% He for 3 × 5 min	Infarct size, K(Ca^2+^) channels activation	He-PC involved mitochondrial uncoupling and induced preconditioning in young but not old rats via K(Ca^2+^) channels	[63]
Young Wistar rats, 2–3 months old, and aged Wistar rats, 22–24 months old (NRW)	Evaluation of age-dependent He-PC on infarct size and role of mito K(Ca^2+^) channels and PKA	He-PC and Age He-PC inhaled 70% He for 3 × 5 min	Infarct size, PKA, mitoK(Ca^2+^) channels	He-PC involved activation of PKA. PKA may be a key mediator for age-dependent loss of preconditioning	[78]
Male Wistar rats, 8–9 weeks old (NRW)	Evaluation of He-PC- and He-PostC on the brain and heart in a rat resuscitation model	Cardiac arrest for 6 min induced by ventricular fibrillation. 70% He and 30% O_2_ for 5 min before cardiac arrest and for 30 min after ROSC	Neurological degeneration, Cognitive function after 7 days, Cav-1, Cav-3 and Hexokinase II in heart tissue	Differential expression levels of Cav-1, Cav-3 and Hexokinase II in the heart, reduced apoptosis in neuronal tissue	[1]
Ex vivo/in vitro					
NRCFs isolated from hearts of 1–3 day old Male Wistar rats (NRW)	Evaluation of He-conditioning angiogenesis, migration, and EV release	Glucose 4 cycles of 95% He + 5% CO_2_ for 1 h, followed by 1-h normoxic condition	Migration, angiogenesis and EV release	He increased fibroblast migration but EVs or soluble factors from cardiac fibroblasts were not involved in the protective effect	[83]
Isolated perfused hearts by 8–12 weeks old C57BL/6 J male mice (NRW)	Evaluation of He late PC on membrane modulation caveolin expression and exosomes	Langendorff performed at 24-h post-He inhalation (30 min)	Levels of Cav-1 and Cav-3 in myocardial tissue, PFP and exosomes	Levels of Cav-1 and 3 were reduced 24 h after He inhalation in whole-heart tissue, and Cav-3 was increased in exosomes from PFP	[214]

7-NI, 7-nitroindazole (selective neuronal NOS inhibitor); AAR, area-at-risk; AG, aminoguanidine hydrochloride (inducible NOS inhibitor); Age He-PC, preconditioning helium aged rats; Akt/PKB, protein kinase B; Ar, argon; Cav-1, caveolin-1; Cav-3, caveolin-3; CINC-3, cytokine-induced neutrophil chemoattractant 3; CO_2_ carbon dioxide; COX-2, cyclooxygenase-2; EPC, early preconditioning; ERK1/2, extracellular signal-regulated kinase 1/2; EV, extracellular vesicle; GSK-3β, glycogen synthase kinase 3; He, helium; 5-HD, 5-hydroxydecanoate; IL-1β, interleukin-1 beta; K(Ca) channels, Ca^2+^-sensitive potassium channel; I/R, ischemia/reperfusion; LAD, left anterior descending coronary artery; L-NAME, N-nitro-l-arginine methyl ester; LPC, late preconditioning; 2-MPG, N-2-mercaptopropionyl glycine; mitoK(Ca) channels, mitochondrial Ca^2+^-sensitive potassium channel; mitoK_ATP_, channels mitochondrial KATP channels; mPTP, mitochondrial permeability transition pore; N_2_, nitrogen; NAAR, non-area-at-risk; NAC, N-acetylcysteine; Ne, Neon; NO, Nitric oxide; NOS, nitric oxide, synthase; eNOS, endothelial NOS; NRCFs, neonatal rat cardiac fibroblasts; NRW, no reported weight; O_2_, oxygen; p70S6K, 70-kDa ribosomal protein s6 kinase; PC, preconditioning; PIF, pifithrin-α; PI3K, phosphatidylinositol-3-kinase; PostC, postconditioning; PFP, platelet-free plasma; PKA, protein kinase A; PKC-ε, protein kinase C ε; RISK, reperfusion injury salvage kinase pathway; ROSC, restoration of spontaneous circulation; ROS, reactive oxygen species; SB21, GSK inhibitor; SHR, spontaneous hypertensive rat; WKY, Wistar Kyoto rat; ZL, Zucker lean rat; ZO, Zucker obese rat

### Argon and cardioprotection

The name argon in Greek: αργός means inert. Argon has anesthetic properties under hyperbaric conditions and is relatively abundant in atmospheric air where it is found at a concentration of 0.93%. It is non-corrosive, non-flammable and non-toxic, with a density 38% higher than that of air and its solubility in water and plasma is 24 times less than that of carbon dioxide [125]. Despite being considered ‘biologically’ inert, recent evidence suggests that argon may have significant pharmacological effects [125, 204]. It has mainly been investigated for its neuroprotective effects but there is some evidence from both in vitro and in vivo studies that Ar exhibited cardioprotective effects and that these effects are mediated by activating ERK1/2 and Akt while regulating c-Jun N-terminal kinases (JNKs) in a biphasic manner [90, 99, 115, 141, 163] (Fig. 3 and Table 5).

### Xenon and cardioprotection

Xenon is regarded as a gaseous anesthetic; despite being costly and scarce, it offers several special benefits such as cytoprotectivity and quick diffusion, along with minimal hemodynamic side effects [38, 169].

The noble gas Xe induces cardioprotection whether applied before (PC-like effect) or after (PostC-like effect) an infarcting ischemia [50, 62, 160, 175, 219]. In particular, in pathological conditions, applying 20% Xe along with 34 ºC hypothermia during early reperfusion can also reduce the area of the MI in rats [175]. Regarding the cardioprotective mechanisms of Xe, it has been suggested that the mitoKATP channel and phosphatidylinositol-dependent kinase-1 are first activated by Xe. These two in turn activate PKC-ε, which in turn activates p38 MAPK. Two downstream targets of p38 MAPK, mitogen-activated protein kinase-activated protein kinase-2 and heat-shock protein 27 (HSP27), are then phosphorylated, which causes HSP27 to translocate to the particulate fraction and increases F-actin polymerization [217–219]. In addition to p38 MAPK, ERK1/2 and cyclooxygenase-2 (COX-2) are critical mediators of either Xe early preconditioning [216] or Xe induced late preconditioning [211]. Xenon can also cause GSK-3β- and Akt-phosphorylation, prevent Ca^2+^ from causing mPTP to open, and maintain mitochondrial function [120].

The minimal side effects of Xe are in line with the minimal effects on cardiac function. Differences were observed between global or regional administration of 50% or 70% Xe in dogs. Regional administration of Xe in the left anterior descending artery only reduced local myocardial contractility when Xe was given at 70% but did not affect global hemodynamics, coronary blood flow and regional myocardial function in the circumflex coronary artery-dependent myocardium [160]. In isolated guinea pig hearts, 40% or 80% of the Xe did not significantly change the NO-dependent flow response, the electrical, mechanical, or metabolic effects. This may be because Xe did not change the major cation currents in the guinea pig cardiomyocytes [196]. Xenon (20, 50, and 65%), in addition to basic intravenous anesthesia, has been demonstrated to cause a reduction in total hepatic O_2_ delivery and venous hepatic O_2_ saturation, as well as a downregulation of heart rate and cardiac output without altering mean arterial pressure or hepatic arterial blood flow [80, 205]. However, it has not been observed to impair intestinal oxygenation in pigs. Actually, pigs with hepatic venous blood that was supplemented with pentobarbital and buprenorphine received 73–78% Xe, which increased the amount of O_2_ in the blood [170]. These data suggest that the effects of Xe on heart rate and hepatic O_2_ levels may be influenced by concomitant intravenous anesthesia.

Since Xe is scarce, expensive, and difficult to administer (only by gaseous route), it is challenging to use Xe outside of controlled respiration and hospital conditions. In addition, it is unlikely to be applied to the management of acute and chronic heart conditions. Researchers have recently solidified Xe, enabling its oral or intravenous administration, by incorporating it into cyclodextrins, starch derivative excipients commonly used as drug carriers [162, 228]. For instance, the saturation point of Xe in water is 0.22 mM only. The solubility of Xe was increased from 0.22 to 0.67 mM when 2-hydroxypropyl-β-cyclodextrin was added as a cage molecule. Supplementing these xenon-enriched solutions by gavage decreased hypertension, left ventricular hypertrophy, and cardiac dysfunction in aged ApoE-knockout mice fed a high-fat diet for six weeks [228]. The acquired information provides opportunities for the creation of medications based on the lyophilized-cyclodextrin-xenon complex that are suitable for transportation, storage, and use in medicine, including outside of hospitals (Table 6).

### Helium and cardioprotection

Helium does not have any anesthetic effects, thus it may be used in awake patients in situations involving ischemia–reperfusion, such as percutaneous coronary interventions for STEMI patients. As He has a low density, it helps patients with airway diseases breath by lowering their energy requirements. Since there are ventilators that enable the application of He via both invasive and non-invasive ventilation strategies, He may also be used in patients undergoing organ transplantation or during heart or vascular surgery. Helium therefore could be a great substitute for Xe or Ar in clinical ischemia/reperfusion scenario because it is a noble gas that is significantly less expensive and there are no known adverse effects of He on regional or global hemodynamics.

Over the last 25 years, several investigations have demonstrated that He has vital cytoprotective properties on endothelial cells [185–187], the brain [1, 23, 31, 56, 104, 107], the gut [36], the liver [232] and the heart [1, 45, 63, 76–78, 127–130, 141, 214].

Helium administration prior to ischemia, known as He preconditioning, exhibits a significant reduction in the infarct size in the model of MIRI, specifically observed in young rats but not in aged rats, and in Zucker lean rats but not in Zucker obese rats [63, 76, 77] (Table 7). The elicitation of helium-induced cardioprotection is associated with the activation of PI3K, MAPK/ERK1/2, p70S6 kinase, cyclic AMP-dependent protein kinase (PKA), COX-2, opioid receptors, mPTP opening, and NO production by eNOS [48, 76–78, 136–141, 215]. In addition, the threshold of helium-induced preconditioning was lowered in vivo by inhibiting GSK-3β or p53 through a mPTP-dependent mechanism [140].

Furthermore, He was cardioprotective when given after ischemia (He-PostC) in the Zucker lean rat or Male Wistar rat models of MIRI. These protective effects on rats are associated with upregulating autophagy-related genes, downregulating apoptosis-related genes [128], raising calcium channel, voltage-dependent, L type (alpha-1D subunit), caveolin-1 and caveolin-3 (Cav-1 and Cav-3) protein levels, and activating ERK1/2 and Akt [45, 77, 128, 215]. According to Smit et al. [186], He preserves post-ischemic endothelial function; eNOS blocking did not reverse this effect.

However, in male adult Wistar rats, a prolonged 30- or 60-min of 70% He dose during reperfusion does not cause cardioprotection [127]. Although the burst of inflammatory cytokines did not decrease, the prolonged inhalation of He may have contributed to the proinflammatory response by raising IL-1β and cytokine-induced neutrophil chemoattractant 3 in the myocardium of the at-risk area, but not in the not-at-risk area [127]. However, when considering the clinical application of noble gases, different approaches have been undertaken for Xe and He. In a study with healthy volunteers, utilizing a forearm blood flow model to explore endothelial function, He was found to alleviate post-ischemic endothelial dysfunction. This improvement occurred without any observed impact on plasma levels of cytokines, adhesion molecules, or microparticles [186]. Furthermore, a clinical study found that neither He-PC (3 × 5 min of 70% He and 30% O_2_ applied before aortic cross-clamping) nor the combination (15 min of He applied before aortic cross-clamp release and continued for 5 min after start of reperfusion;) had any effect on the activation of p38 MAPK, ERK1/2, or the levels of PKC-ε and HSP27 in the hearts of patients undergoing coronary artery bypass grafting (CABG) procedures. Moreover, in these patients, postoperative troponin release was unaffected by helium-pre- or helium-postconditioning [184].

For Xe, it was shown, that unlike hypothermia alone, the addition of Xe to hypothermia reduced myocardial damage in patients following out-of-hospital cardiac arrest and return of spontaneous circulation [8]. The combination of Xe with hypothermia was linked to a more substantial recovery of left ventricular systolic function compared to hypothermia alone. This suggests that Xe possesses cardioprotective properties in this clinical context [173].

However, a multinational, randomized clinical trial examined the cardioprotective effects of Xe anesthesia in patients undergoing CABG surgery, comparing it to anesthesia based on sevoflurane or propofol. Among the 492 patients who received either propofol, sevoflurane, or Xe, a decrease in troponin I release was observed in the Xe group compared to the propofol group, as well as in the sevoflurane group compared to the propofol group. However, the difference in troponin release was minimal, and it remains uncertain whether this observed effect holds clinical significance [71]. In line with these results, a study in patients with American Society of Anesthesiologists physical status III undergoing aortic surgery under either Xe or total intravenous anesthesia did not find significant differences in global myocardial performance, myocardial contractility or laboratory values between the groups [13]. Furthermore, in a randomized trial in 30 patients who underwent elective on-pump CABG receiving balanced anesthesia of either Xe or sevoflurane, Xe rather triggers proinflammatory effects and suppresses the anti-inflammatory response [17]. The clinical relevance of these findings, however, has not been determined in this study.

Taking these results into account, even though the noble gases can be easily applied in the clinical situation, their cardioprotective effects so far have not yet been strongly supported by larger randomized trials. Therefore, additional studies are necessary to clarify these discrepancies between experimental and clinical studies. For instance, the presence of a threshold in the hypertensive heart is suggested by the fact that, in spontaneous hypertensive rats, only a triple intervention of He-PostC can reduce MIRI, in contrast to the healthy Wistar Kyoto rats [129]. According to an in vitro study He-conditioning enhanced fibroblast migration, but not the release of extracellular vesicles of protective medium or soluble factors from the cardiac fibroblasts, contributed to cardioprotection [83]. According to a recent study, a dose-dependent improvement in lipopolysaccharide-induced left ventricular dysfunction and cavity enlargement can be obtained with intraperitoneal injection of 99.999% He; the optimal dose, in this study, was found to be 1.0 ml/100 g [234]. In terms of mechanisms, in this study, He decreased the phosphorylation of NF-κB, inhibited the expression of toll-like receptor 4 and then attenuated the expression of TNF-α and IL-18 (Table 7).

## Interactions between gasotransmitters and noble gases

From the preceding discussion, it can be inferred that noble gases frequently act on the same targets as gasotransmitters, either directly or indirectly, leading to the induction of endogenous gas formation and signaling pathways activation. The synergistic interactions between gasotransmitters and noble gases in cardioprotection scenario have become a focal point of scientific exploration [6, 7, 9, 139], and recent studies have delved into the experimental intricacies surrounding their collective impact. Notably, investigations suggest that these gases protect endothelial cells [209, 211, 212] and collaboratively induce vasorelaxation within the cardiovascular system, bolstering their individual and synergistic cardioprotective effects. As seen above, this phenomenon has been examined in controlled experimental settings, employing various in vitro and in vivo models of MIRI. Experiments involving perfused hearts, isolated vessels, and animal models have provided valuable insights into the specific mechanisms through which the interplay of NO, H_2_S, CO, and noble gases—Ar, He, and Xe in particular—manifests as a shield against MIRI (Tables 1, 2, 3, 4, 5, 6, and 7) [209, 231].

As seen above, the metabolic alterations observed in cardiovascular system cells during chronic illnesses (comorbidities) and their response to hypoxia are intricately linked with the involvement of endogenous gasotransmitters as well as with exogenous gases, including noble gases. As concern gasotransmitters, we have mentioned that they are generated as part of metabolic processes. For instance, NO is produced during the breakdown of arginine to citrulline, H_2_S is formed during cysteine metabolism, and CO is a byproduct of heme metabolism. The reader is redirect to recent reviews for the discussion on how these metabolic processes contribute to cardioprotection, specifically regarding gasotransmitters in the context of comorbidities [5–7, 9, 148, 180, 239]. These gaseous molecules play pivotal roles in modulating vascular tone, promoting angiogenesis, and ensuring cellular survival against hypoxic conditions and oxidative stress induced by IR. As seen, their effects are mediated via the modulation of signaling pathways that influence mitochondrial function and metabolism, and the activity of key regulators of intracellular processes, including but not limited to PKG, PI3K, MAPK, JNK1/2, sGC, cGMP, NF-κB, HIF-1α, as well as ion channels such as mitoKATP channels and large conductance calcium-activated potassium (BK_Ca_) channels (Figs. 2 and 3). Indeed, BK_Ca_ channels are paradigmatic in elucidating the interaction and cardiovascular protective effects of gasotransmitters. Gases activating BK_Ca_ channels may confer protective effects and reduce vascular resistance during episodes of hypoxia and MIRI [180]. BK_Ca_ channels are target of H_2_S on cardiomyocytes under hypoxic conditions as well as upon endogenous CO stimulation of cardiomyocytes. Carbon monoxide modulates the function of these channels by interacting with reduced heme. Nitric oxide triggers the activation of BK_Ca_ channels indirectly through pathways associated with PKG and PKA signaling. Cellular functions related to Ca^2+^ storage and energy synthesis heavily rely on the capacity of these channels across cell and organelle membranes. Indeed, effective strategies to mitigate MIRI include activating energetic metabolic pathways such as glycolysis, glucose and ketone oxidation, as well as hexosamine biosynthesis, and deacetylation. Yet, protection can be achieved by reducing the activity of the malate-aspartate shuttle, mitochondrial O_2_ consumption, fatty acid oxidation, and mitochondrial succinate metabolism, requiring novel reversible and specific inhibitors. Currently, the most promising metabolic therapy for MIRI seems to involve targeting glycolysis, O-GlcNAcylation, and the metabolism of ketones, fatty acids, and succinate, either individually or in combination [239]. We have reported above the impact of gases on mitochondrial function and O_2_ consumption. Moreover, evidence suggests that their involvement in cellular metabolism extends to regulating energetic substrate utilization [131, 146, 169]. For instance, tyrosine residue nitration in mitochondrial key proteins contributes to regulate mitochondrial and cellular function after MIRI [239]. Consequently, the combination of gasotransmitters holds the potential to modulate metabolism, mitigate mitochondrial damage, and ultimately reduce MIRI. The crosstalk between gasotransmitters and noble gases has been probed at the molecular level shedding light on potential molecular targets for advanced cardioprotective strategies [181, 188, 213]. Controlled studies utilizing gasotransmitter-releasing compounds and precise administration protocols have demonstrated the therapeutic promise of these molecules in mitigating MIRI [82, 133]. Similarly, experiments employing various delivery methods for noble gases, including inhalation and controlled release, have showcased their organ-protective properties with a focus on detailed dose–response relationships and pharmacokinetics [227].

In conclusion, the ongoing research in the intricate interplay between gasotransmitters (NO, H_2_S, SO_2_, CO) and noble gases (Ar, He, Xe) paves the way for novel therapeutic strategies, underlining the potential of these molecular shields against the global health challenge of ischemic heart disease.

## Nanodevices to deliver gases

Nanoparticle-based drug delivery has gained significant popularity as a strategy to optimize the therapeutic potential of drugs. Despite notable improvements, formulating gases presents unique challenges not encountered with liquid and solid active ingredients. The challenges and failures of gases-oriented therapies in the field of cardioprotection have been recently and extensively reviewed [6, 7, 9, 146]. In brief, drug formulation and tolerance as well as comedications and comorbidities may affect the outcomes [44, 92]. Specifically, in comorbidities, elevated production of ROS/RNS can influence cardiac gases-dependent signaling and induce adaptive changes in the expression and activity of enzymes producing gases as well as antioxidant enzymes. Initially, these changes may serve as protective mechanisms in metabolic syndrome and diabetes [148]. However, prolonged exposure to oxidative, nitrosative, and nitrative stress can deplete these protective mechanisms, leading to increased ROS/RNS production and reduced gases bioavailability (especially for NO and H_2_S) in the myocardium. Furthermore, heightened oxidative and nitrosative stress can impair the NO-sGC signaling pathway, limiting the ability of NO to perform its essential signaling functions in the heart. The upregulation of ROS/RNS production in the presence of risk factors also promotes the activation of redox-dependent transcription factors like NF-κB, which stimulates the expression of various proinflammatory mediators, contributing to the development of cardiac dysfunction and remodeling. The dysregulation of gases-dependent signaling may affect the therapeutic effectiveness of conventional drugs used in managing metabolic syndrome, hypertension and diabetes. Conversely, modulation of gases-dependent signaling could underlie the therapeutic benefits of established and newly developed treatment approaches, such as ACE inhibitors, specific β-blockers, and sGC activators as well as NO or H_2_S donors conjugated with other pharmacological agents such as non-steroidal anti-inflammatory drugs [6, 7, 9, 114, 146] A deeper understanding of these pathological processes and pharmacology of gas-enhancers holds promise for developing more effective therapeutic strategies to combat comorbidities and its cardiac consequences related to gases-dependent signaling. Gas molecules or noble gases, released from formulations for therapeutic purposes, have been studied, although insufficiently. Gasses can be modified into prodrugs called gas-releasing molecules (GRMs) and subsequently released from these GRMs. For instance, the development of CORMs stemmed from the need for compounds capable of transporting and releasing controlled amounts of toxic CO within cellular systems. This approach offered a promising avenue for studying the pharmacological effects of the gas and elucidating its mechanisms of action [82, 133, 157]. Extensive coverage is given to various nanosystems and their roles in efficient shuttling, targeting, and release of therapeutic gases. Diverse ways have been proposed in which GRM prodrugs, in delivery nanosystems, respond to intrinsic and extrinsic stimuli for sustained release, offering a comprehensive overview of their design and application in nanomedicine [133]. Considerable investigation has been conducted into the utilization of inorganic materials for the loading and delivery of drugs. These include, photothermal agents [100, 102, 233], photon‐to‐photon conversion agents such as lanthanide [237], catalytic metal ionic species [111], photocatalytic nanomaterials [210, 236] and polymer‐based nanomaterials [162, 176, 224, 228], to name only a few used for gas-loading in different pathological conditions (Table 8). In a related context, recent developments introduce two types of O_2_-loaded nanodevices (ND) based on alpha-cyclodextrin and cyclic nigerosyl-nigerose. These NDs demonstrate protection against hypoxia/reperfusion-induced cell death in vitro, serving as biocompatible and biodegradable drug carriers for simultaneous O_2_ and therapeutic delivery [43, 150]. Advances in solidifying Xe within cyclodextrins make oral or intravenous administration feasible [162, 228]. Hence, O_2_- NO- and/or Xe-loaded NDs, conjugated with an ischemic myocardium-targeting peptide (that is protein/peptide‐modified materials), could efficiently deliver to the infarcted heart. This methodology involves synthesizing ND formulations that integrate O_2_, gasotransmitters, and/or specific noble gases, achieved through thoughtful modifications around the glucose ring to enhance efficiency. In addition, lipid nanoparticles have proven effective in drug delivery and play a significant role in controlling the release and diffusion of gases within biological tissues in gasotransmitter applications [133]. Several stimuli-responsive GRMs have been tested for therapeutic gas release, emphasizing the importance of efficient and targeted delivery at diseased sites. Intrinsic stimuli, such as pH, offer high biocompatibility but demonstrate weaker driving forces for gas release compared to external stimuli such as magnetic, ultrasound, and near-infrared light (NIR)-triggers. Controllable external stimuli generally exhibit high release efficiency and specificity. Combining intrinsic and external stimuli can enhance responsiveness. Critical challenges for intrinsic stimuli include the need for improved sensitivity. External stimuli, such magnetic, ultrasound, and NIR triggers due to their superior characteristics might be preferred (Table 8). However, external stimuli may cause tissue irritation at high powers [133].

**Table 8:** Overview of nanodevices charged with gases

Nanomaterials	Gas	Release strategy	Therapeutic effects	Additional features	Refs.
*Polymeric nanomaterials*					
*Synthetic polymers* (**e.g.**, PEG, PLA, PCL, PLGA and HPMA) and *natural polymers* (**e.g.**, albumin, gelatin, cyclic nigerosy-nigerose chitosan, dextran, cyclodextrins and heparin)	O_2_ CO NO H_2_S Xe	Spontaneous release, influenced by pH or glutathione	Limiting cardiomyoblasts hypoxia/reoxygenation injury in vitro. Cardiovascular protection in ex vivo and in vivo settings	So far, the most studied are water-soluble cyclodextrins, and cyclodextrin-based nanosponges providing extended and controlled gas release, as well as mPEG–PLGA. Encapsulation of different gases to improve permeation, stability and slow gas release	[43, 133, 150, 162, 176, 224, 228]
*Photothermal agents*					
Bi_2_S_3_ and BNN	NO	NIR light irradiation	So far, photothermal therapy has been used in oncological diseases	BNN employed for NO-enhanced mild photothermal therapy upon 808 nm irradiation	[133, 233]
Reduced graphene oxide	NO H_2_S	NIR light irradiation	So far, elevated temperature (> 45 °C), to induce cancer cell apoptosis in oncological diseases	Polyethyleneimine-dithiocarbamate was employed as H_2_S prodrug assembled on reduced graphene oxide nanosheet	[100, 133]
Mesoporous Prussian blue	CO	NIR light irradiation	So far, used to elevate tumor temperature (> 50 °C), CO release, mitochondrial oxygen consumption, overcoming multidrug resistance	Incorporation of pentacarbonyl iron as CORMs, less-invasive photothermal effect	[102, 133]
*Photon-to-photon conversion agents*					
Lanthanide upconversion nanoparticles	NO CO	Absorb NIR lights to emit UV–Vis photons	So far, used for NO release in a light dosage-dependent manner to increase cell penetration. CORM prodrug delivery system for targeted cancer gas therapy	Gas releasing molecules are more sensitive to light in the UV–vis region than in NIR region	[133, 237]
*Catalytic metal ionic species*					
Cu(0)-nanoparticle plus hydrophilic polyurethane	NO	Reduction of Cu	Cu(I) form via Cu(II) reduction by thiolate anion, leading to RS-Cu^+^ intermediate. Subsequently, Cu(I) binds to the nitrogen and releases NO to prevent platelet aggregation	Substance encapsulated: S-nitroso-N-acetylpenicillamine. Intravenous infusion	[111, 133]
*Photocatalytic nanomaterials*					
Photocatalytic nanomaterial (**e.g.**, HisAgCCN)	CO	630 nm laser irradiation	Endogenous CO_2_ conversion to CO; so far, used for cancer therapy	Histidine-rich peptides were utilized to modify carbon-dot-decorated C_3_N_4_ nanoparticles doped with Ag_3_PO_4_, creating a metal–organic framework-based photocatalytic material	[133, 236]
Partially oxidized nanosheets	CO	561 and/or 808 nm laser irradiation	Endogenous CO_2_ conversion to CO; so far, used for cancer therapy	Tin disulfide (nanosheets were partially oxidized) and subsequently integrated with doxorubicin within the nanosystem	[133, 210]

Bi_2_S_3_, bismuth sulfide; BNN, N,N′-Di-sec-butyl-N,N′-dinitroso-1,4-phenylenediamine; CO, carbon monoxide; CO_2_, carbon bioxide; CORMS, CO‐releasing molecules; Cu, copper; H_2_S, hydrogen sulfide; HPMA, N-(2-hydroxypropyl)-methacrylamide copolymer; NIR, near-infrared light; NO, nitric oxide; O_2_, oxygen; PCL, poly(ε-caprolactone); PEG, poly(ethylene glycol); mPEG, methoxyPEG; PLA, poly(lactic acid); PLGA, poly(lactic-co-glycolic acid); UV, ultraviolet; Xe, xenon

While there have been a few clinical trials conducted on the gasotransmitter H_2_S (refer to Table 1 in [9]), a multitude of studies have explored NO donors, nitrates, and nitrites, which are beyond the scope of this review. In particular, evidence from clinical practice indicates that H_2_S may produce inconclusive outcomes in patients with cardiovascular diseases. A clinical trial investigating the impact of Na_2_S on patients with acute STEMI was withdrawn by the researchers (NCT01007461), while another study, known as the Groningen Intervention study for the Preservation of cardiac function with sodium thiosulfate after STEMI (GIPS-IV; NCT02899364), demonstrated neutral findings [30]. To our knowledge, CO is often studied as toxic agent and only one trial has been conducted thus far involving CO in patients with stable chronic obstructive pulmonary disease (NCT00122694). Overall, further experimentation is crucial to advance the GRMs and/or stimuli for potential clinical applications in gasotransmitter and noble gases research. Nevertheless, the synthesis and testing of these formulations are deemed essential, particularly in the cardiovascular field and relevant myocardial infarct models, providing a promising avenue for future clinical applications, recognizing the imperative of adopting a multitarget approach in addressing MIRI [29]. This strategy underscores the potential efficacy of exploiting gaseous diffusibility as a readily attainable means of achieving multifaceted therapeutic goals.

## Conclusions

In summary, we highlight the diverse yet interconnected roles of gasotransmitters and noble gases in modulating molecular pathways to promote cell survival and overall tissue health. While gasotransmitters such as NO and H_2_S, produced by various cell types, contribute significantly to various physiological processes, noble gases such as He and Xe emerge as promising candidates for organ protection. Their ability to modulate molecular pathways and promote cell survival underscores their cardioprotective potential. However, collaborative efforts between experimental and clinical researchers are essential to bridge the gap between laboratory findings and real-world applications. Prospective studies may focus on establishing optimal dosages, timings, and combination therapies involving both noble gases and gasotransmitter donor molecules. Integrating advanced imaging techniques, molecular profiling, and systems biology approaches will contribute to a comprehensive understanding of the intricate molecular pathways involved. Of course, it is crucial to acknowledge the influence of cardiovascular disease, confounders and risk factors on endogenous cardioprotective mechanisms. Here, we highlight the need to address the impact of cardiovascular comorbidities on the regulation of gasotransmitters and noble gases, as well as the challenges associated with the clinical application of their cardioprotective potential [5, 44, 154, 184, 187]. Moving forward, incorporating information on the effects of cardiovascular risk factors on individual gasotransmitters and exploring new avenues for therapeutic strategies will be essential for advancing the field and optimizing patient outcomes. Research in various areas has emphasized the significance of interactions among gasses, suggesting the need for integrated investigation into their role in MIRI and cardioprotection. Gasses influenced pathways intersect in common downstream effectors during processes such as cardioprotection, vasodilation and angiogenesis. Therefore, effective therapeutic approaches may require the utilization of multiple gases or their pharmacological modulators, considering also their potential in affecting cell metabolism [9, 131, 146, 169, 239]. This multidimensional approach is crucial for developing more effective and targeted cardioprotective strategies.

## Data Availability

This is a review article, and therefore, there are no original data to be made available.
